# Stable enhancers are active in development, and fragile enhancers are associated with evolutionary adaptation

**DOI:** 10.1186/s13059-019-1750-z

**Published:** 2019-07-15

**Authors:** Shan Li, Evgeny Z. Kvon, Axel Visel, Len A. Pennacchio, Ivan Ovcharenko

**Affiliations:** 1 Computational Biology Branch, National Center for Biotechnology Information, National Library of Medicine, National Institutes of Health, Bethesda, MD 20892 USA; 2 Environmental Genomics and Systems Biology Division, Lawrence Berkeley National Laboratory, Berkeley, CA 94720 USA; 3 United States Department of Energy Joint Genome Institute, Walnut Creek, CA 94598 USA; 4 School of Natural Sciences, University of California, Merced, CA 95343 USA; 5 Comparative Biochemistry Program, University of California, Berkeley, CA 94720 USA

**Keywords:** Enhancer, Evolution of gene regulation, Transcription factor interaction, Causal regulatory variants, Transgenic mouse reporter assay

## Abstract

**Background:**

Despite continual progress in the identification and characterization of trait- and disease-associated variants that disrupt transcription factor (TF)-DNA binding, little is known about the distribution of TF binding deactivating mutations (deMs) in enhancer sequences. Here, we focus on elucidating the mechanism underlying the different densities of deMs in human enhancers.

**Results:**

We identify two classes of enhancers based on the density of nucleotides prone to deMs. Firstly, fragile enhancers with abundant deM nucleotides are associated with the immune system and regular cellular maintenance. Secondly, stable enhancers with only a few deM nucleotides are associated with the development and regulation of TFs and are evolutionarily conserved. These two classes of enhancers feature different regulatory programs: the binding sites of pioneer TFs of FOX family are specifically enriched in stable enhancers, while tissue-specific TFs are enriched in fragile enhancers. Moreover, stable enhancers are more tolerant of deMs due to their dominant employment of homotypic TF binding site (TFBS) clusters, as opposed to the larger-extent usage of heterotypic TFBS clusters in fragile enhancers. Notably, the sequence environment and chromatin context of the cognate motif, other than the motif itself, contribute more to the susceptibility to deMs of TF binding.

**Conclusions:**

This dichotomy of enhancer activity is conserved across different tissues, has a specific footprint in epigenetic profiles, and argues for a bimodal evolution of gene regulatory programs in vertebrates. Specifically encoded stable enhancers are evolutionarily conserved and associated with development, while differently encoded fragile enhancers are associated with the adaptation of species.

**Electronic supplementary material:**

The online version of this article (10.1186/s13059-019-1750-z) contains supplementary material, which is available to authorized users.

## Background

Analysis of genomic variants in humans and model species such as mouse and fruit fly is providing opportunities for understanding the genetic basis of complex traits and disease predisposition. The vast majority of complex trait-associated variants from the Genome-Wide Association Studies (GWAS) are located in non-coding regulatory regions—only approximately 5% of GWAS SNPs are located in the protein-coding regions [1, 2]. Accumulating evidence suggests that genetic variants within regulatory sequences may alter the binding of TFs to induce gene expression variation and ultimately result in complex phenotypic changes [3–9]. However, recent genome-wide studies reveal that variable TF-DNA binding is not only driven by sequence alterations in the motif of the cognate TF [10–12], but additional features such as the nucleotide composition of motif-neighboring sequences, the chromatin context of a genuine binding site, and the three dimensional (3D) structural confirmation of DNA, which are also important to model TF-DNA binding [13–17]. For example, in mouse mature white adipose tissue, PPARγ bindings that vary between close strains do not harbor an altered motif but exhibit linkage to extensive co-localization with motifs corresponded to CEBPα and glucocorticoid receptor [18, 19].

To accurately identify the causal regulatory variation, advanced and flexible machine learning models, such as support vector machine (SVM) [5, 15] and deep learning neural network [13, 20], have been constructed by integrating versatile features associated with enhancer activity. These models are commonly trained on multiple layers of genomic, epigenomic, and transcriptomic information. One of these advanced approaches, an SVM-based method CellulAr-dePendent dEactivating mutations (CAPE) developed by us [15], has been demonstrated to be accurate in predicting causal regulatory variants which either mediate the chromatin accessibility (CAPE dsQTL model) or impact gene expression (CAPE eQTL model). Its methodology stems from the general appreciation that the motif alone cannot accurately predict differential TF binding or enhancer activity and, therefore, should be complemented with supplemental information obtained from the sequence environment and chromatin features around the focal variant. The causal regulatory variants predicted by CAPE are termed deactivating mutations (deMs).

Employing CAPE to the enhancer regions in hepatocellular carcinoma (HepG2) cells, we observed a large spectrum of density of putative deactivating mutation positions (dubbed deMPs) across the panel of these enhancers. Enhancer deMP nucleotide content ranges from 0.04 to 36%. On average, approximately 1.5% of enhancer positions are deMPs and thus are prone to deactivating effects. This startling difference in deMP density motivated us to investigate the inherent mechanisms of the regulatory programs of the enhancers with different densities of deMPs, such as inter-TF interactions and evolutionary constraints. After categorizing HepG2 enhancers to three sets (fragile, regular, and stable) based on their densities of deMPs, we observed that the deMP densities are negatively correlated with the functional and evolutionary stability of enhancers. Fragile enhancers have elevated deMP densities that make them prone to a wide range of single-nucleotide deactivation mutations and, thus, make it harder to preserve their function during evolution. This kind of enhancer is specific to the defense system and cellular maintenance and might benefit from the panel of deMPs in them, which might represent a pathway for evolutionary innovation and adaptation. By contrast, stable enhancers are impervious to single-nucleotide deactivations and more functionally conserved. This set of HepG2 enhancers is strongly associated with liver development and regulation of transcription factor activity. In addition, the two sets of enhancers have different cohorts of TFBSs and benefit from different modes of inter-TF interactions. The homotypic TFBS cluster usage provides the stable enhancers with more buffering once a mutation takes place. Additionally, we observed that the mode of TF interaction and multiple layers of chromatin context contribute more to the binding site sensitivity to one single-nucleotide deactivation, as compared to the cognate motif itself.

By applying transgenic mouse reporter assay of two stable enhancers in human heart left ventricle, we observed that lacZ expression of the transfected enhancers was abolished (or diminished) in the mouse embryonic hearts due to the top 5% mutations identified by our algorithm, which substantiate the functional impact of the predicted deactivating mutations and further support the rationale of the two classes of enhancer activities.

## Results

### Enhancers with different densities of deMPs evolve under different evolutionary constraints

**Fig. 1:**
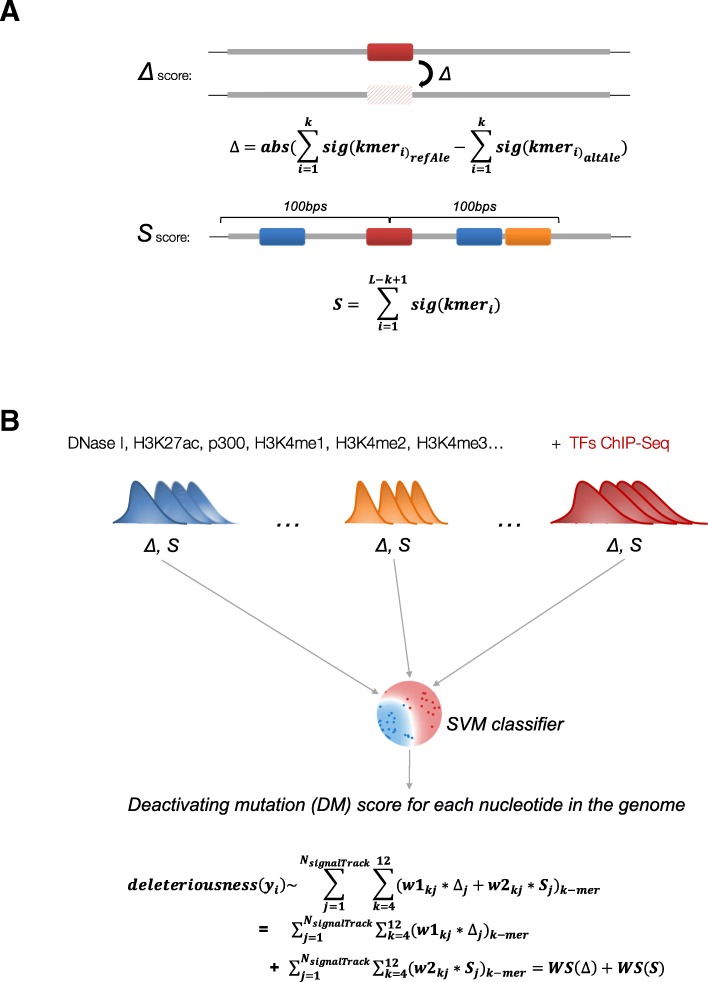
The two factors of a CAPE score. **a** The two factors associated with each genetic variant in the classifier: Δ, the disruptive effect on the cognate motif caused by a single-nucleotide variant; *S*, the binding capability of the neighboring region. **b** The general idea of the CAPE—integration of two factors (Δ and *S*) learned from variable enhancer-associated ChIP-seq peaks and DNase to the SVM classifier. Refer to formula 2 in the “Methods” section for the details of parameters

**Fig. 2:**
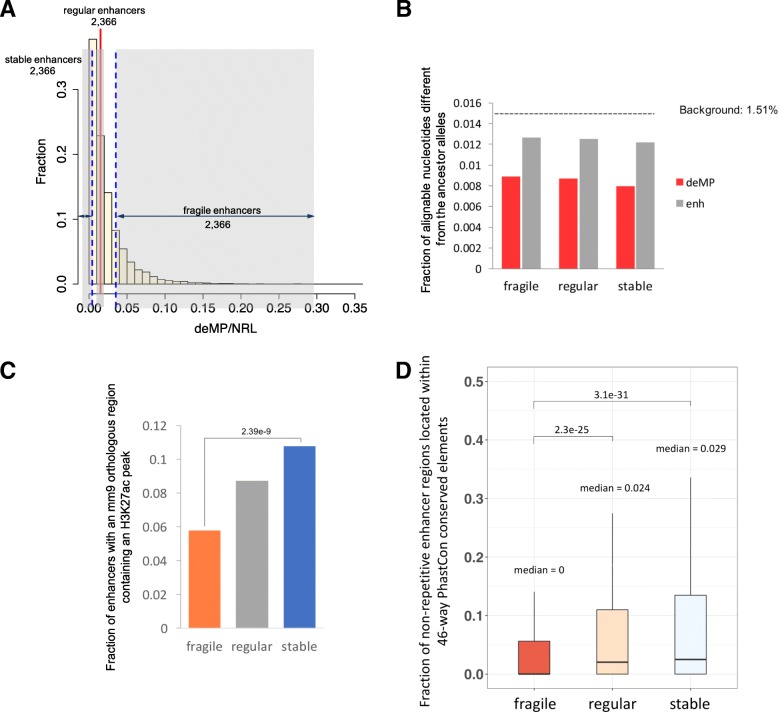
Enhancers with distinct densities of deMPs in HepG2 cell line have different functional constraints. **a** Distribution of densities of deMPs in HepG2 enhancers. The top, middle, and bottom 20% of enhancers correspond to fragile, regular, and stable enhancers, respectively. **b** deMPs are under strong purifying selection compared to enhancer regions. The *y*-axis shows the fraction of alignable nucleotides different from the chimp genome. The dashed line labels the expected fraction of alignable nucleotide different from the chimp genome using pseudogenes as the background. **c** More orthologous sequences of stable enhancers in the mouse genome have H3K27ac peaks than those of fragile enhancers. The *p* value was calculated based on Fisher’s exact test. **d** Larger fraction of stable enhancers located within phastCon conserved elements compared to fragile enhancers. *p* values were calculated using the Mann–Whitney–Wilcoxon test

The goal of our study was to investigate the association between the density of causal regulatory variants in an enhancer, the DNA sequence composition, and the evolutionary constraint of that enhancer. To identify the *cis*-regulatory causal variants and examine their distribution in HepG2 cell line, we applied the SVM classifier CAPE [15], with the eQTL model (using random SNPs with matched minor allele frequency and similar distance to the nearest TSS as the negative control set), to predict mutations that are likely to affect gene expression and deactivate enhancers (see the “Methods” section). The basic idea behind CAPE is to utilize a learned enhancer-associated sequence code to infer the deleterious effect of a potential regulatory genetic variant on enhancer activity. This approach integrates two characteristics associated with a genomic variant—the ability of a variant to disturb the cognate TF binding event and co-binding of other TFs in the neighborhood. These two sequence signatures were learned from a panel of enhancer-associated signals, including DNase-seq, H3K27ac, H3K4me1, H3K4me2, H3K4me3, H2A.Z, P300, and major TF binding data from the corresponding tissue [15] (Fig. 1). In other words, the predicted deleteriousness of a genetic variant is composed of a weighted linear combination of the two signatures under a variety of enhancer-associated chromatin profiles. Therefore, a CAPE score can be further partitioned into two parts: a weighted linear combination of the binding affinity change across all signals [denoted as WS(Δ)] and a weighted linear combination of the neighborhood binding capabilities across all signals [denoted as WS(*S*)] (see the “Methods” section, Fig. 1). WS(Δ) and WS(*S*) represent the overall contribution of the binding affinity change and interactions of combinations of TFs within the surrounding sequence, respectively, to the impact on enhancer activity. The mutations identified by CAPE (FPR ≤ 0.01) were dubbed deactivating mutations (deMs), as they are likely to disrupt the binding of an essential TF and lead to a deleterious impact on the enhancer activity and, in some cases, result in enhancer deactivation. The genomic positions holding at least one deM were dubbed deMPs. In general, this approach is limited to mutations that are observed frequently and that have matching expression observations, in the relevant tissue or cell type. For the tissues without eQTL information, this method is not directly applicable. Overall, 167,303 deMPs reside in approximately 57% (11,831/20,936) of HepG2 active enhancers. We then focused on the active enhancers with at least one deMP in their sequence. On average, approximately 1.5% of enhancer positions are deMPs (Fig. 2a). For 80% of the enhancers, the deMP sequence content is less than 3.6%. Therefore, in general, deMPs are sparsely distributed along enhancer regions. The enhancers that correspond to the long tail of the deMP density distribution are the exceptions that feature up to 36% of deMP nucleotide content (Fig. 2a).

To test how deMPs in enhancers relate to the target gene function and the evolution of gene regulation in liver, we classified our set of HepG2 enhancers (*n* = 11,831) into three categories based on their deMP density: fragile enhancers (*n* = 2366, top 20%, 6.3% deMP nucleotides), regular enhancers (*n* = 2,366, middle 20%, 1.5% deMP nucleotides), and stable enhancers (*n* = 2,366, bottom 20%, 0.25% deMP nucleotides) (Fig. 2a, Additional file 1: Figure S1). We speculated that the mutations occurring at deMPs might disrupt enhancer activity, while numerous deMPs would make an enhancer fragile, i.e.*,* more prone to deactivating mutations. Specifically, enhancers with more deMPs could lose their function during evolution faster compared to the ones with fewer deMPs. We primarily focused on two extreme sets of enhancers (stable and fragile) in this study. To investigate the relationship between enhancer fragility and deMP density, we first assessed the evolutionary constraints of deMPs by computing the percentage of the positions different from the chimp genome and the fraction of positions carrying single-nucleotide polymorphisms (SNPs). Overall, deMPs are under stronger purifying selection than regular, non-repetitive enhancer nucleotides (Fig. 2b and Additional file 1: Figure S2), which is consistent with our previous observations [6]. Consequently, mutations at these highly constrained sites might have a stronger deleterious impact on enhancer activity than regular enhancer regions. Next, to compare the level of functional conservation of all sets of enhancers, we mapped the three categories to mouse liver enhancers and observed that, although a similar fraction (approximately 30%) of HepG2 enhancer sequences of the three categories have orthologous counterparts in mouse (Additional file 1: Figure S3), stable enhancers have more active mouse orthologous enhancers relative to fragile enhancers (Fig. 2c). By further mapping the three sets of enhancers to cow, dog, and opossum, we consistently observed that stable enhancers are more likely to preserve their regulatory activity during mammalian evolution as compared to fragile enhancers (Additional file 1: Figure S4). We also examined the overall functional constraints of all sets of enhancers using placental mammal phastCon conserved elements. Fragile enhancers display a significantly lower degree of sequence conservation as compared to stable enhancers (phastCons scores at non-repetitive DNA regions; Fig. 2d). Fragile enhancers are also less conserved at the functional level (as measured by the fraction of the human/mouse enhancer orthologs that are active as enhancers in the same tissue in both species). To investigate whether the lower functional conservation of fragile enhancers is largely associated with the multiplicity of deMPs apart from their lower degree of sequence conservation, we subsampled enhancers with similar sequence constraints between the two classes and compared their degree of function conservation. Stable enhancers are significantly more conserved at the functional level compared to fragile enhancers, independent of the degree of sequence conservation (Additional file 1: Figure S5). All the observations above suggest that the high density of deMPs makes the fragile enhancer more prone to single-nucleotide deactivating mutations and, therefore, harder to retain its regulatory function during evolution.

### Stable and fragile enhancers are associated with distinct biological pathways

**Fig. 3:**
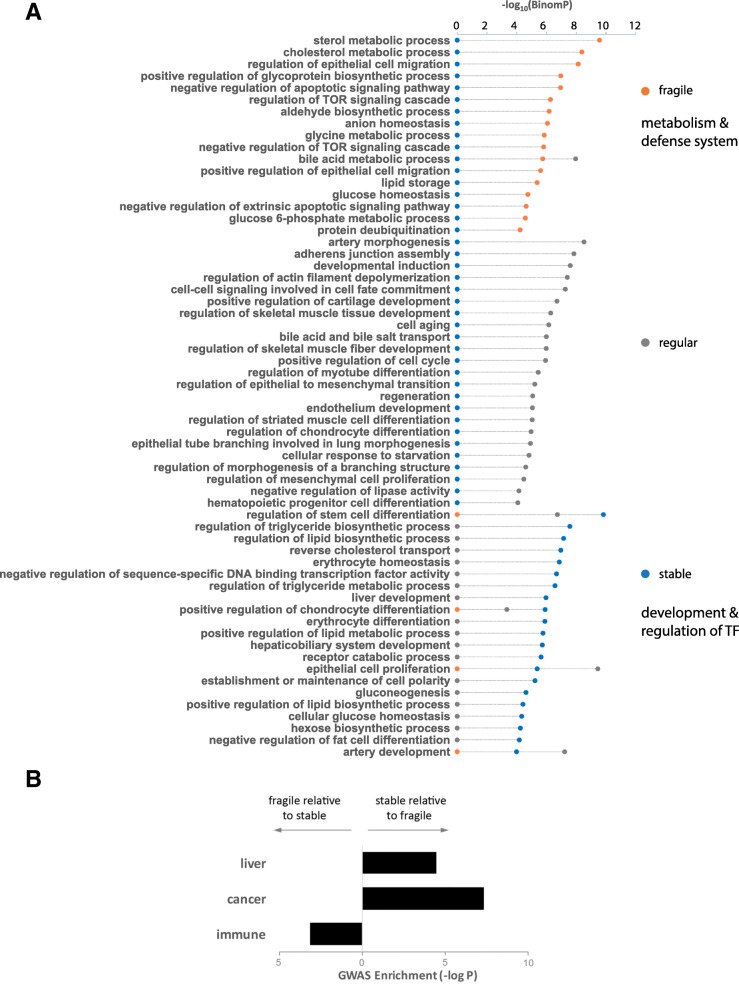
Different densities of deMPs in HepG2 enhancers yield distinct functional enrichment. **a** Fragile and stable enhancers are associated with different biological pathways. Results were obtained using the programming interface for the online enrichment tool GREAT version 3.0.0 [21]. **b** Enrichment significance (hypergeometric distribution testing) of different disease types in fragile and stable enhancers

Since the deMP density is associated with the evolutionary constraint of enhancers, we conjectured that fragile enhancers and stable enhancers are likely to regulate different groups of genes. To examine the biological functions of the enhancers with various deMP densities, we applied the annotations enrichment tool GREAT [21] to functionally characterize the two categories of enhancers. The fragile enhancers are primarily associated with metabolic processes and the defense system-related signaling pathways, including the target of rapamycin (TOR) signaling cascade and apoptosis (Fig. 3a). TOR is a conserved Ser/Thr kinase that regulates cell growth and metabolism in response to various environmental cues, such as growth factors, nutrients, energy, and stress [22]. By contrast, the stable enhancers are specifically associated with the development and regulation of transcription factor activity (Fig. 3a).

Next, we analyzed the enrichment of GWAS traits in the two sets of enhancers to further determine if enhancers with distinct densities of deMPs are associated with different human diseases and/or phenotypic traits. The fragile enhancers are enriched in traits of various immune diseases such as type I diabetes, IgG glycosylation (part of the human humoral immune response), rheumatoid arthritis, and multiple sclerosis. This suggests a dominant role of fragile enhancers in the defense system to a greater extent compared with stable enhancers (Additional file 2: Tables S1-S4 and Fig. 3b). The notion that the human-specific immune responses are emphasized by genes involved in apoptosis, as well as by genes associated with susceptibility to infectious diseases or immune-related disorders [23], further suggests the role of fragile enhancers in the immune system. Moreover, the weaker functional constraint on the fragile enhancers is also accordant with the rapid evolution of the immune system [23]. In contrast, the stable enhancers are strongly associated with liver-related traits such as metabolic syndrome (blood metabolic levels, blood metabolic ratios), blood lipid levels (LDL peak particle diameter, adiponectin levels, visceral adipose tissue adjusted for BMI, visceral adipose tissue, subcutaneous adipose tissue ratio), and glucose homeostasis traits (Additional file 2: Tables S1-S4 and Fig. 3b). Notably, stable enhancers are also significantly associated with traits of various cancers (Fig. 3b), suggesting that regulatory variants of developmental systems could lead to detrimental phenotype change, such as cancer.

### Different regulatory sequences encoding in fragile and stable enhancers

**Fig. 4:**
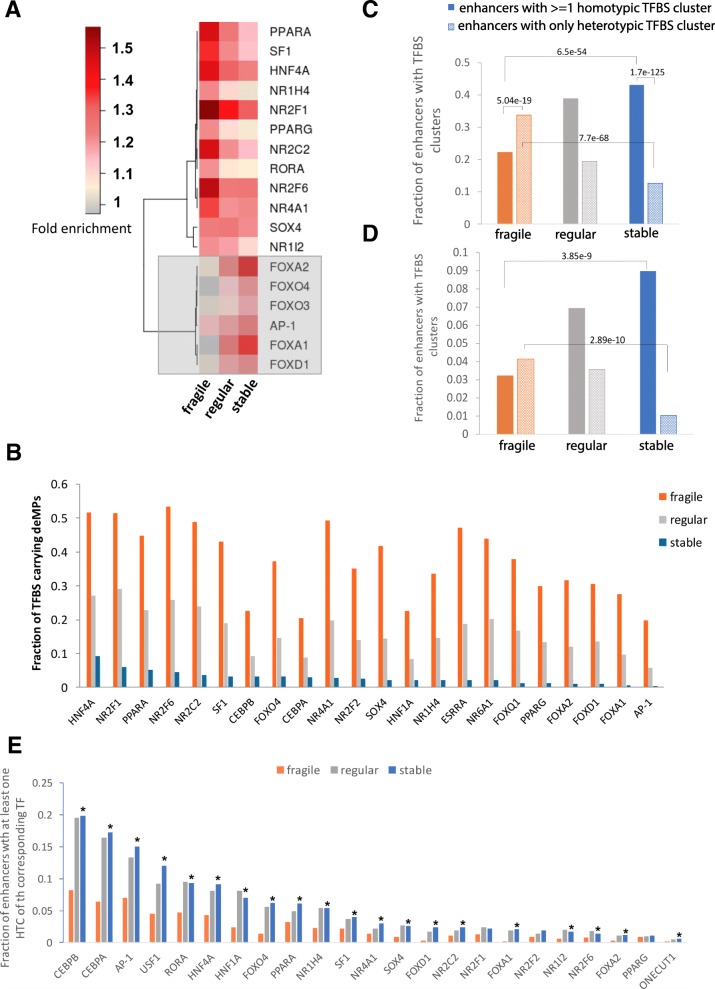
Fragile and stable enhancers employ different regulatory codes. **a** Different cohorts of TFBSs are enriched in enhancers with different densities of deMPs. The legend shows the range of fold enrichment (see the “Methods” section) of TFBSs in a set of enhancers relative to their negative control. **b** TFBSs in fragile enhancers are prone to one single-nucleotide deactivation, while TFBSs in stable enhancers are impervious to one single-nucleotide deactivation. **c** Homotypic TFBS clusters are more enriched in stable enhancers, whereas heterotypic TFBS clusters are more enriched in fragile enhancers. Only the TFBSs shown in Fig. 4a were included in the analysis. **d** Functional homotypic TFBS clusters are more enriched in stable enhancers, whereas functional heterotypic TFBS clusters are more enriched in fragile enhancers. Only the TFBSs located within corresponding ChIP-seq peaks and shown in **a** were included in the analysis. **e** Liver-specific TFBSs are more likely to form homotypic TFBS clusters (HTCs) in stable enhancers as compared to fragile enhancers. The asterisk indicates the Fisher exact test *p* value < 0.01

The two categories of enhancers have remarkably distinct deMP densities, which promoted us to conjecture that fragile enhancers might have much larger TFBS densities than stable enhancers, considering that deMPs deactivate enhancers through disrupting TF binding. To address this question, we first identified the potential TFBSs enriched in the two sets of enhancers. Interestingly, the two categories of enhancers are bound by different classes of TFs. The motifs of the FOX family of pioneer TFs, including FOXA1, FOXA2, FOXD1, FOXO3, and FOXO4, are enriched in the stable enhancers and not fragile enhancers. By contrast, the motifs of the nuclear receptor family, consisting of HNF4A, PPARA, NR2C2, NR4A1, NR2F6, PPARG, NR1H4, RORA, and NR1I2, are enriched in the fragile enhancers to a larger extent as compared to the stable enhancers (Fig. 4a). The enrichment of the in vivo transcription factor binding in the three classes of enhancers is consistent with the enrichment of in silico predicted TF motifs. Specifically, the stable enhancers are enriched for ChIP-seq peaks of FOXA1, FOXA2, and AP-1 (subunits: c-JUN and FOSL2), whereas the fragile enhancers show enrichment of nuclear receptor factors HNF4A, HNF4G, and NR2F2 (Additional file 1: Figure S6). Notably, the well-known regulatory roles of the enriched TFs are quite accordant with the associated biological functions of the corresponding enhancer category. In particular, the initiation of liver development is dependent on FOXA transcription factors [24]. Other than that, the FOXA clade has been shown to function in many developmental processes in multiple tissues [25]. Therefore, the exclusive enrichment of TFBSs of the FOXA clade in stable enhancers further corroborates a strong contribution of stable enhancers to liver development. In addition, the FOXO proteins are not only essential mediators of glucose homeostasis but also crucial tumor suppressors—major mediators of the activation of the PI3K and Akt signaling pathways in cancer [25, 26], which is accordant with the enrichment of GWAS cancer traits in the stable enhancers relative to fragile enhancers (Fig. 3b). As for the nuclear receptors whose binding motifs are enriched in fragile enhancers, they have been extensively studied and identified to regulate lipid and glucose metabolism, bile acid homeostasis, drug disposition, inflammation, and various aspects of tissue repair, including liver regeneration and fibrosis [27], further supporting the role of fragile enhancers in the immune system and cellular maintenance.

We next examined the densities of these enriched TFBSs in the two categories of enhancers. Surprisingly, the two sets of enhancers have very similar densities of enriched TFBSs (Additional file 1: Figure S7), so that TFBS densities might not be the cause of the different deMP densities. Therefore, it could be either the different TFBS composition or different inter-TF interactions that lead to the distinct deMP densities. We observed that both TF families have an astonishingly different percentage of binding sites overlapping a deMP in the two sets of enhancers: 20–50% of TFBSs enriched in fragile enhancers host a deMP compared to 0.4–9% of enriched TFBSs in stable enhancers hosting a deMP (Fig. 4b), further excluding the different TFBS composition as the underlying paradigm. Consequently, the striking difference in the deMP density in these two categories of enhancers could be explained if the deleterious effect of a single-nucleotide mutation occurring at one site is buffered by the presence of one or more compensatory sites of the same TF in an enhancer. Indeed, homotypic TFBS clusters of liver-specific TFs are enriched in stable enhancers relative to fragile enhancers, while heterotypic TFBS clusters of liver-specific TFs are more abundant in fragile enhancers (Fig. 4c). To further accurately quantify the TF-TF cooperativity pattern, we identified TFBS clusters (homotypic and heterotypic) using only the TFBS motifs residing within their corresponding TF ChIP-seq peaks. Our analysis shows that the stable enhancers are almost exclusively enriched for homotypic TFBS clusters, whereas heterotypic TFBS clusters dominate fragile enhancers (Fig. 4d). This trend is consistent with the cooperativity patterns based on all potential TFBS motifs (Fig. 4c). On the other hand, homotypic TFBS clusters are depleted of deMPs as compared to other TF binding sites, although deMPs tend to target the binding sites of essential liver-specific transcription factors compared to other enhancer regions (Additional file 1: Figure S8), suggesting that deMs primarily stem from mutations disrupting the binding of essential transcription factors or co-factors, whereas the enrichment of homotypic TFBS clusters may alleviate the impact of single-nucleotide changes on TF binding or enhancer activity. Therefore, the stable enhancers enriched with homotypic TFBS clusters are likely to be depleted of deMPs compared to fragile enhancers. It has been shown in a previous study [28] that homotypic TFBS clusters are key components of developmental enhancers and play an important role in the regulation of transcription factors, which, again, substantiate the role of development and regulation of transcription factor activity of stable enhancers.

### TF interaction modes and chromatin contexts contribute more to the fragility of TF binding than the cognate motif

Since the TFBSs enriched in fragile enhancers are prone to deMPs, whereas the TFBSs in stable enhancers are impervious to deMPs (Fig. 4b), we next studied what contributed more to the different extents of the fragility of TF binding in the two sets of enhancers, namely, the cognate motif or interactions among TFs?

**Fig. 5:**
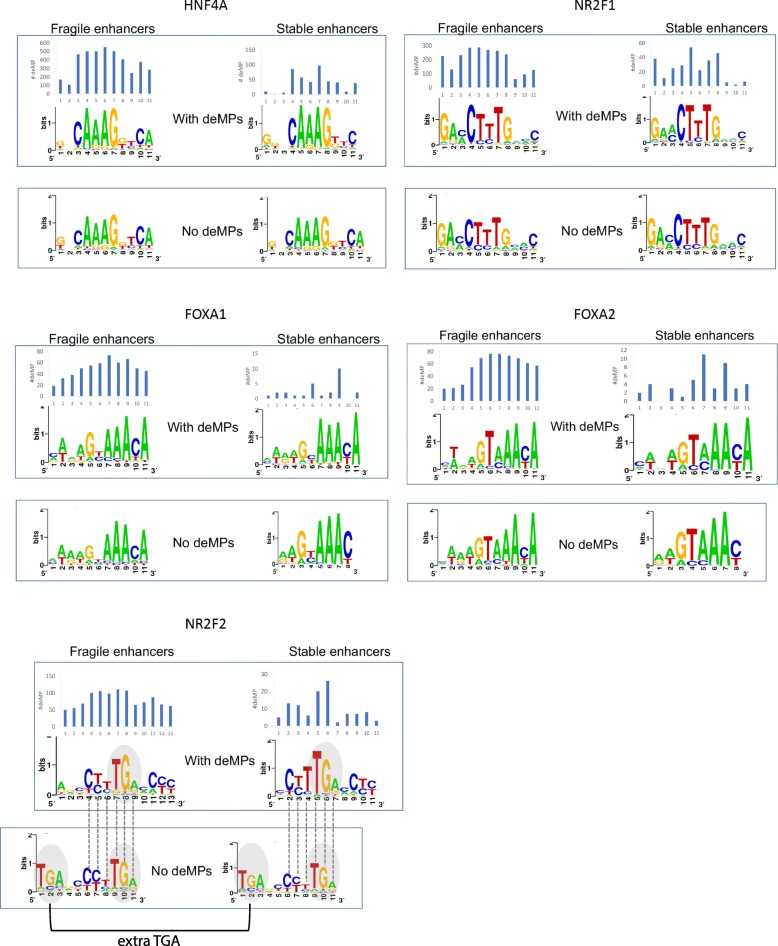
Multiple TFs have two variants of binding sites—prone and impervious to a single mutation deactivation. The histogram above the motif prone to deMPs shows the distribution of the relative positions of deMPs along the motif. The motifs were generated using WebLogo [29]

**Fig. 6:**
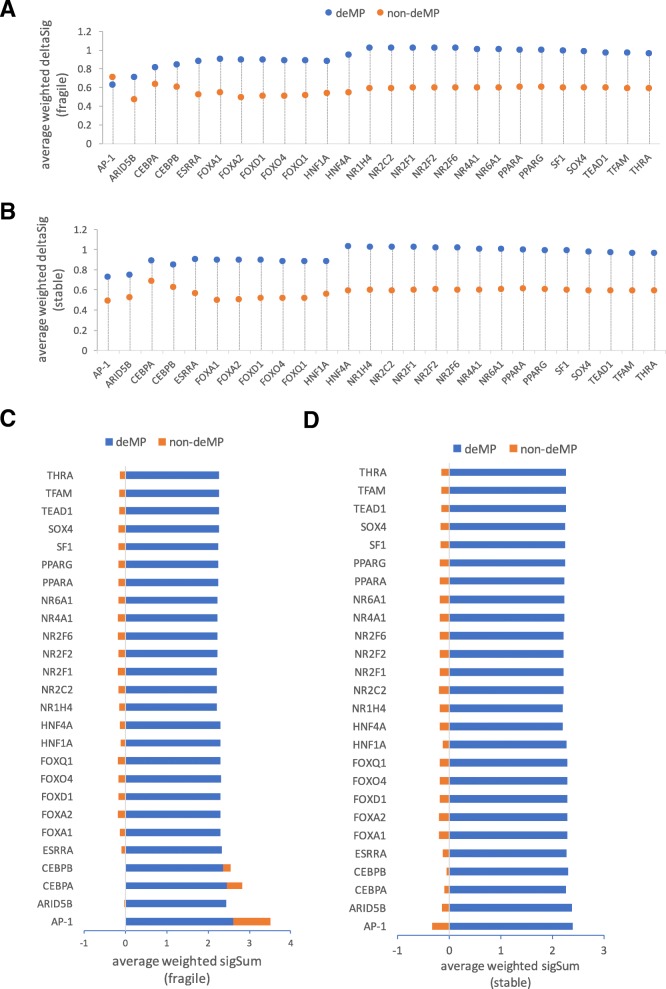
TF collaboration mode and chromatin landscape contribute more to the fragility of TF binding as compared to the cognate motif. Comparison of the mean of WS(Δ) of the motif variants with the maximum CAPE score between the motifs prone to deMP and the ones impervious to deMP in **a** fragile enhancers and **b** stable enhancers. Comparison of the mean of WS(*S*) of the motif variants with the maximum CAPE score between the motifs prone to deMP and the ones impervious to deMP in **c** fragile enhancers and **d** stable enhancers

We first asked whether the two variants of TF binding, prone or impervious to a single mutation deactivation, employ different motifs. As shown in Fig. 5, for the majority of TFs, the two forms of binding sites utilize very similar motifs. The exception is NR2F2. NR2F2 has an extra TGA in the motif of the binding sites impervious to deMP, which might help mitigate the deleterious effect of a single-nucleotide mutation. As for the majority of TFs, due to their usage of similar cognate motifs for the two variants of TF binding, we hypothesize that the direct or indirect interactions between the combinations of TFs in the immediate vicinity or the larger chromatin landscape largely influence the fragility of TF binding and enhancer activity. To address this question, we decomposed the CAPE score of a genetic variant to the two factors, namely, the weighted linear combination of binding affinity change across all signals [denoted as WS(Δ)] and the weighted linear combination of the neighborhood binding capabilities under all chromatin profiling [denoted as WS(*S*)] (see the “Methods” section), to estimate which is the more important factor governing the fragility of TF binding. Specifically, we compared the two factors of a genetic variant in the binding site prone to deMP with those of a genetic variant in the binding site impervious to deMP to ascertain which factor contributes more to enable a mutation to deactivate an enhancer. For the binding sites hosting either at least one or no deMP, the genetic variant with the highest CAPE score within a binding site was selected and decomposed to WS(Δ) and WS(*S*). The deMP with the highest CAPE score within a binding site was denoted as max-deMP, and the mutation with the highest CAPE score within a binding site not overlapping deMPs was denoted as max-non-deMP. By sequentially taking the mean of WS(Δ) and WS(*S*) over the max-deMPs (or max-non-deMPs) across all the binding sites of each TF, we observed that although the binding affinity change caused by the max-deMP is noticeably larger than that caused by max-non-deMP (Fig. 6a, b), the interaction of TFs in the immediate vicinity or the larger chromatin context is likely to be a more important factor that governs the fragility of TF binding and enhancer activity (Fig. 6c, d).

### CAPE predicts functional impact of enhancer variants

**Fig. 7:**
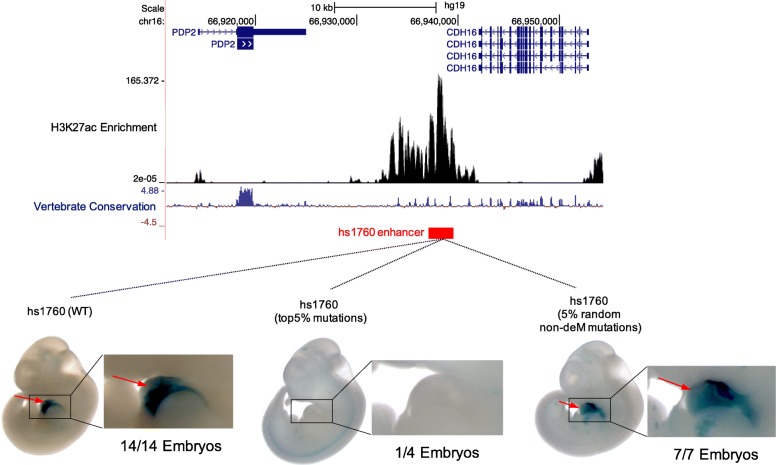
CAPE accurately predicts the effect of mutations with the highest CAPE scores on heart enhancer activity in vivo. Schematic of the human hs1760 heart enhancer locus is shown on the top. Enhancer activity of three versions of hs1760: hs1760 wild type (WT), hs1760 with top 5% mutations including the deMs, and hs1760 with 5% random non-deM mutations in transgenic E11.5 mouse embryos are shown. Both the wild-type hs1760 enhancer sequence and hs1760 with 5% non-deM random mutations could drive LacZ expression in E11.5 mouse heart (red arrow); by contrast, the top 5% mutations including deMs could deactivate this enhancer in E11.5 mouse heart. Numbers of embryos with lacZ activity in the heart over the total number of transgenic mouse embryos screened are indicated. The graphic of the genomic coordinates with the track of sequence conservation (phyloP scores) was obtained from UCSC genome browser (http://genome.ucsc.edu/; UCSC hg19 assembly). The H3K27ac enrichment data of the human heart left ventricle was obtained from ROADMAP epigenomics project [32]. The image of the wild-type hs1760 was obtained from VISTA Enhancer Browser (https://enhancer.lbl.gov/). See Additional file 1: Figures S9-S10 for details

To directly test the ability of CAPE to predict the functional consequence of sequence variation on enhancer activity in vivo, we turned to two stable enhancers active in the human heart (left ventricle) (see the “Methods” section) and tested the functional impact of deMs using the transgenic mouse reporter assay. We selected these two enhancers (hs1760 and mm69; Additional file 2: Table S5) based on the following criteria: (1) both enhancers show strong H3K27ac enrichment in the left ventricle of the human heart; (2) both of them belong to the evolutionarily conserved regions (ECRs) between human and mouse/rat identified by the ECR browser (https://ecrbrowser.dcode.org/) [30]; and (3) either the enhancer itself or its mouse ortholog has strong lacZ signal in mouse heart from VISTA Enhancer Browser (http://enhancer.lbl.gov) [31] (Fig. 7, Additional file 1: Figures S9-S10). To test if the predicted deMs have a deleterious effect on enhancer activity, we compared the lacZ reporter activity at mid-gestation (embryonic day [E] 11.5) between the enhancer with the top 5% mutations with the highest CAPE scores and those with the 5% random non-deM mutations. The top 5% mutations indeed disrupted the activity of hs1760 enhancer—only one out of four mouse embryos showed weak lacZ staining in the heart. Conversely, the 5% random non-deM mutations did not affect hs1760 enhancer activity: all the seven transgenic mouse embryos displayed lacZ staining in the heart, four of which showed strong lacZ expression (Additional file 1: Figure S9 BC). As for the second enhancer, a human ortholog of mm69, although its LacZ expression was not fully abolished after introducing the top 5% mutations, the lacZ expression was diminished in the majority of the six embryos—only embryo #4 showed strong LacZ expression. In contrast, two out of four mouse embryonic hearts exhibited strong lacZ expression due to the introduced 5% non-deM mutations (Additional file 1: Figure S10 BC). The attenuated lacZ activity caused by the top 5% mutations at mm69 ortholog indicates that the LacZ expression is gone in some but not all heart cells, which can also corroborate the deleteriousness of the mutations with the highest CAPE scores. We speculated that the failure of the introduced deMs to completely abolish the activity mm69 is possibly due to a false-positive prediction of CAPE, which to some extent might be caused by the slightly different regulatory codes between human and mouse, whereby the predicted deMs in human may not totally disrupt the binding of the same transcription factor in mouse.

## Discussion

In this study, we focused on elucidating the mechanism that underlies the different densities of deactivating mutations in HepG2 enhancers. Due to the high functional constraint on deMPs, fragile enhancers are prone to single-nucleotide deactivation and, therefore, are more likely to lose their regulatory function faster than stable enhancers during evolution. In contrast, stable enhancers are impervious to single-nucleotide deactivations and more functionally conserved. The fragile enhancers are strongly associated with the defense system and metabolism, whereas the stable enhancers are mainly enriched in the development and regulation of transcription factor activity. The distinct evolutionary constraints of enhancers with different densities of deMPs correlate well with their corresponding biological functions: genomic variants in fragile enhancers are often associated with immune system disorders based on GWAS studies, yet the enriched traits in stable enhancers are more liver-related and also include more cancers, suggesting that the consequences of mutations that disturb development or transcription factor activity are often fatal. Emera et. al. proposed a model of enhancer evolution in the neocortex [33]. They conjectured that the enhancers likely emerge as proto-enhancers (short sequences with low regulatory information content), some of which serve as nucleation points for evolving to complex enhancer cores that contain more regulatory information under constraint. The enhancer cores further evolve to composite enhancers composed of ancient and derived functional segments. Therefore, the older enhancers are often longer and contain more functional sites. Accordant with their observations, although the lengths of host gene loci have a similar distribution between the two sets of enhancers (Additional file 1: Figure S11), the stable enhancers are longer (Additional file 1: Figure S1) and harbor more functional elements (Figs. 2d and 4a) as compared to the fragile enhancers. This further suggests a stronger evolutionary constraint on the development-related enhancers (stable enhancers) compared to the immune system-related enhancers (fragile enhancers). In addition, stable enhancers exhibit stronger enhancer activity compared to fragile enhancers, in terms of H3K27ac ChIP-seq signal intensity, liver-specific transcription factor binding ChIP-seq signal intensity, and in vitro regulatory activity measured by Sharpr-MPRA (Additional file 1: Figure S12). Possibly, this is a consequence of the larger number of functional and homotypic binding sites in stable enhancers, which results in elevated stability of bound proteins and a diminished likelihood of transient binding.

In deciphering the regulatory code of these two sets of enhancers, we observed that binding sites of different families of TFs are enriched in fragile and stable enhancers. The pioneer factors from the FOX family are exclusively enriched in stable enhancers, as opposed to the stronger enrichment of the nuclear receptor family TFs in fragile enhancers. Interestingly, the TFs enriched in stable enhancers exhibit a lower level of tissue specificity compared to those enriched in fragile enhancers (Additional file 1: Figure S13), which is consistent with the evolutionary resilience of ubiquitously expressed genes in the mammalian liver [34]. In addition, we observed a substantial difference in the fraction of TFBSs prone to deMP in fragile and stable enhancers: up to 50% of TFBSs in fragile enhancers contain at least one deMP, while, on average, approximately 5% of TFBSs in stable enhancers contain deMPs. The huge difference in the fragility of TFBSs in these two sets of enhancers prompted us to further investigate the collaboration mode of TFBSs. Remarkably, the stable enhancers are more likely to employ homotypic TFBS clusters. This built-in redundancy of TFBSs in stable enhancers may buffer genetic perturbations that affect one of the motifs. Therefore, the grammar of stable enhancers appears to be consistent with the billboard model, in which the positioning of TF binding sites is flexible and subject to loose distance, and only a subset of TFBSs are required to be active at a given time [35, 36]. By contrast, the fragile enhancers are more likely to harbor heterotypic TFBS clusters and may approximate the enhanceosome model, in which the DNA motif composition and relative positioning act as a scaffold to cooperatively recruit all TFs, forming a higher-order protein interface to regulate transcription [36, 37]. In this scenario, all transcription factors that bind to an enhancer might be essential for the cooperative occupancy and activation of an enhancer. On the other hand, the usage of different TFBS collaboration modes in the two sets of enhancers may also correspond to two alternative fashions to open the chromatin. Without the binding of pioneer factors to open the chromatin, the fragile enhancers have to recruit combinations of TFs to compete with nucleosomes to bind the DNA, sometimes in a manner of heterotypic TFBS clusters. In this regard, loss of binding of a TF due to one single-nucleotide mutation might also lead to loss of binding of other synergistically bound TFs, inducing the deactivation of the enhancer. Conversely, the stable enhancers are enriched with the pioneer factor FOXA. Different from other enriched liver-specific TFs of stable enhancers, FOXA barely binds the enhancer in a mode of homotypic TFBS clusters (Fig. 4e). Without multiple compensatory sites, the binding sites of FOXA that are prone to single-nucleotide deactivation would have easily lost their function during evolution. Therefore, most FOXA binding sites are impervious to deMs in stable enhancers (Fig. 4b), possibly by using a less constrained motif with shorter informative core motif compared to the FOXA binding sites that are more sensitive to deMs to some extent (Fig. 5).

More importantly, we find that TFBS interaction and multiple layers of chromatin context contribute more to binding site fragility as compared to the cognate motif. As such, the majority of TFs can recognize regulatory regions based not only on pure sequence information of the motif, but also on the chromatin landscape of the DNA at neighboring sequences around the focal motif variant. However, further experimental validation is needed to unveil and interpret versatile binding patterns of different TFs and even higher-order interaction between TFs and chromatin. Other than that, alternative mechanisms such as differential DNA repair might not be ruled out to contribute to the drastically different fragility of the two sets of enhancers, which would need more comprehensive studies. Finally, by applying the targeted mutagenesis mouse reporter assay to two enhancers, we validated the deleterious functional impact of the predicted deMs on enhancer activity in vivo. The two enhancers exhibited abolished/diminished enhancer activity after introducing the top 5% mutations. The detrimental impact of the mutations identified by CAPE, based on the transgenic reporter mouse assay, further corroborated the dichotomy of enhancer activity. In short, with the stable enhancers at the foundation for development and transcription factor activity and with the fragile enhancers linked to an adaptable regulatory program for the defense system and cellular maintenance, this bimodal system contributes to the fitness and adaptation of species. Nevertheless, the cancerous nature of HepG2 cell line precludes us from fully generalizing our observations to enhancers active in primary cells.

## Conclusions

Quantifying perturbations in gene regulation caused by non-coding mutations and associating such mutations with phenotypic changes are a challenging task. Not only non-coding mutations are often silent (with no impact on TF-DNA binding), but also not all non-coding mutations affecting TF binding have a measurable impact on gene expression. Here, we identified the mutations that are likely to deactivate TF binding and investigated the sensitivity of enhancers to single-nucleotide mutations. The fragile enhancers with abundant deactivating mutation nucleotides are associated with the immune system and regular cellular maintenance, whereas the stable enhancers with only a few deactivating mutation nucleotides are associated with development and regulation of transcription factors and are evolutionarily and functionally conserved. These two classes of enhancers encoded by different regulatory programs have contrasting levels of tolerance of deactivating mutations and employ different modes of TF-TF interactions. We found that TF-TF interactions, and not individual TF-DNA binding events, are the key contributor to enhancer deactivating mutations. Our study profiles deleterious mutations in human enhancers and identifies specific enhancer nucleotides most sensitive to mutations, thus providing an atlas of non-coding mutations likely linked to disease susceptibility and evolutionary innovations. However, versatile binding patterns of different TFs and interaction between TFs of these two classes of enhancers await further elucidation.

## Methods

### Data access

#### Identification of deMPs

The general idea of identification of deMP is first to identify the candidate deactivating mutations that disrupt a putative binding site and next to score these candidate deactivating mutations using CAPE, a tool we developed to identify causal regulatory variant in enhancer regions [15]. All three possible mutations at a genomic position, regardless of whether they exist as human SNPs, were scored by CAPE. The mutations with significant CAPE scores were considered to be deactivating mutations. The genomic positions holding at least one deactivating mutation were dubbed deactivating mutation positions (deMPs).

Specifically, we utilized the *k*-mer vocabularies trained on ChIP-seq enhancers to infer the sequence specificities of TFBSs. The enriched *k*-mers (*k* = 8) were assumed to be the potentially functional TFBSs [6] on ChIP-seq enhancers. To identify the enriched *k*-mers in HepG2 enhancers, we first generated a set of controls for each enhancer sequence. Controls were randomly sampled from the whole genome with the same GC-content, repeat-content, and length as the corresponding enhancer sequence. Five control sequences were extracted for each enhancer. In cases when not enough controls with our strict criteria (ΔGC-content ≤ 0.005, Δrepeat-content ≤ 0.01) could be identified, we created additional controls by reshuffling enhancer sequences. For each of the possible 32,896 *k*-mers (*k* = 8), we used the Fisher exact test to evaluate the enrichment of *k*-mers in the HepG2 enhancer set and identified the top 522 *k*-mers significantly enriched in enhancers (*p* ≤ 1e−3 after the Bonferroni correction) as potentially functional *k*-mers and 30,647 insignificant *k*-mers (*p* > 1e−3 without the Bonferroni correction) as background *k*-mers.

As we did in our previous study [6], we applied a modified intragenomic replicates (IGR) model [38] to recognize candidate deactivating mutations that change a top *k*-mer to a background *k*-mer once we identified top *k*-mers in the positive training set. The candidate deactivating mutations were next scored by CAPE. For the HepG2 cell line, only the mutations with significant CAPE scores (CAPE score ≥ 0.57156, corresponding to FPR ≤ 0.01) were considered to be deMs. We used the change of the associated *k*-mers to identify the candidate deactivating mutation before applying CAPE due to the limitation of the CAPE score. The output of CAPE is the probability of a mutation being a causal regulatory variation by either decreasing or increasing the enhancer activity. Since we are particularly focused on mutations deactivating enhancers, we need to limit the candidate deactivating mutations to the ones that could disrupt a potential binding site using the *k*-mer vocabularies.

To identify the deMPs in the left ventricle, we trained CAPE on the human left ventricle eQTLs by integrating the regulatory signals of this tissue (H3K27ac, H3K4me1, H3K4me3, P300, DNase, H3K36me3, H3K27me3, H3K9me3). We then scored all possible single-nucleotide variants (SNVs) in the left ventricle enhancer region. Only the mutations with CAPE score ≥ 0.58276 (FPR ≤ 0.01) were identified to be deMs (Additional file 1: Figure S14). The top 20% of enhancers with the most abundant deMPs correspond to fragile, and the bottom 20% of enhancers devoid of deMPs correspond to stable enhancers, respectively. The top 5% mutations with the highest CAPE scores and 5% random mutations of the two stable enhancers (hs1760 and human ortholog of mm69) are listed in Additional file 2: Tables S6-S7.

### Functional enrichment analysis using GREAT

Functional enrichment of enhancers was performed using the online Genomic Regions Enrichment of Annotations Tool (GREAT) version 3.0.0 [21]. In the GREAT figure (Fig. 3a), the default distance parameter was applied for the regulatory domain assignment of genes, and the single nearest gene rule was applied to associate enhancers with genes. The Gene Ontology (GO) biological process terms were included only if they satisfied the strict criteria in at least one category of enhancers: (1) binomial *p* value ≤ 1e−4, (2) a minimum binomial observed region hits and hypergeometric observed gene hits of 10, and (3) a minimum binomial region and hypergeometric gene set fold enrichment of 2. The −log_10_ binomial *p* values were plotted on the *y*-axis. To show that the GO enrichment of both fragile and stable enhancers is robust by different gene association rules, the other two gene association options (“basal plus extension” and “two nearest genes”) were also applied (Additional file 1). To compensate for the bias caused by assigning all the enhancers to their nearest genes, 45% of enhancers were randomly relocated before applying GREAT for 10 times (Additional file 1).

### Enrichment analysis of GWAS traits

The NHGRI GWAS Catalog was downloaded in September 2016 [1]. The GWAS traits that coincided with the single-nucleotide polymorphisms (SNPs) of the three sets of enhancers were first grouped by disease type (Additional file 2: Table S4). To study the enrichment of a set of SNPs coinciding with a certain disease type, the tag SNPs coinciding with the GWAS traits were further expanded by linkage disequilibrium (LD) (*r*^2^ > 0.8, maximum distance of 500 kb). The enrichment of stable enhancer SNPs coinciding with a disease type relative to fragile enhancer SNPs was evaluated as −logP of the hypergeometric distribution, and vice versa.

### Identification of potential TFBSs in the three sets of enhancers

For the purpose of identifying the location of potential binding sites, we used the profiles of binding sites for vertebrate TFs stored in Jaspar [39], CIS-BP [40], SwissRegulon [41], HOCOMOCO [42], and UniPROBE [43] databases. We trained an in-house developed tool called tfbsFrag on random sequences to create optimized position-specific scoring matrices (PSSM) identified by FIMO [44] to maintain the rate of false-positive discoveries in a real genomic sequence to about five false positives in 10 kb of sequence. We then used tfbsFrag and the optimized vertebrate PSSMs to scan the enhancer sequences of the three classes. The human reference genome hg19 was hard-masked to eliminate the transposable elements when searching for potential TFBSs. Five random sequences were generated for each enhancer sequence with strict criteria (ΔGC-content ≤ 0.005, Δrepeat-content ≤ 0.01), which were used for PSSM identification and for determining the TFBSs enrichment of an enhancer set relative to the background. The occurrence of a particular TFBS in a set of enhancer/random sequence was normalized by the total length of non-repetitive enhancer/random regions. Then, the enrichment of the TFBSs of TF A (i.e., TFBS_A_) in a set of enhancers is determined by formula 1.

1 $$ \mathrm{Enrichment}=\frac{\frac{\#{\mathrm{TFBS}}_{\mathrm{A}}\ \mathrm{in}\ \mathrm{a}\ \mathrm{set}\ \mathrm{of}\ \mathrm{enhancer}}{\mathrm{total}\ \mathrm{length}\ \mathrm{of}\ \mathrm{non}-\mathrm{repetitive}\ \mathrm{enhancer}\ \mathrm{region}}}{\frac{\#{\mathrm{TFBS}}_{\mathrm{A}}\ \mathrm{in}\ \mathrm{a}\ \mathrm{set}\ \mathrm{of}\ \mathrm{control}\ \mathrm{sequences}}{\mathrm{total}\ \mathrm{length}\ \mathrm{of}\ \mathrm{non}-\mathrm{repetitive}\ \mathrm{control}\ \mathrm{region}}} $$

If an enhancer harbored at least three potential binding sites for a TF expanding no more than 1 kb, we assumed that this enhancer had at least one homotypic TFBS cluster. Analogously, if an enhancer harbored at least three potential binding sites for different TFs expanding no more than 1 kb, we assumed that this enhancer had at least one heterotypic TFBS clusters.

### Partition CAPE score

CAPE is a support vector machine-based classifier aimed at predicting causal regulatory variant [15]. In brief, it learns sequence code from large-scale chromatin profiling data of multiple signal tracks, including DNase-seq, H3K27ac, H3K4me1, H3K4me2, H3K4me3, H2A.Z, P300, and major TF binding data of the corresponding tissue. Two sequence signatures, namely, the disruptive effect of the mutation on major TF binding (Δ) and the co-binding of TFs in its neighborhood (*S*), are the basic component of features for each signal (Fig. 1a). In all, CAPE integrates (*N*_*k*_ × *N*_*k*merSignature_ × *N*_signalTrack_) features. *N*_*k*_ (= 5) is the number of *k*-mer sizes (*k* = 4, 6, 8, 10, 12). *N*_*k*merSignature_ (= 2) is the number of signatures including the binding affinity change of the potential binding site due to the mutation (Δ) and the overall binding capabilities of the nearby sequence context of the genetic variant (*S*). *N*_signalTrack_ is the number of the chromatin data (Fig. 1b). The optimal weights for the features learned from the fivefold cross-validation of the eQTL model of HepG2 cell line [15] are listed in Additional file 2: Table S8. The optimal hyperplane of the classifier can therefore be partitioned to two components—the weighted sum of disruptive effect on the cognate motif (denoted as WS(Δ)) and the weighted sum of the co-binding of other TFs in the flanking region (denoted as WS(*S*)) (formula 2).

2 $$ \mathrm{Deleteriousness}\left({y}_i\right)\sim \sum \limits_{j=1}^{N_{\mathrm{signalTrack}}}\sum \limits_{k=4}^{12}{\left(w{1}_{kj}\ast {\Delta }_j+w{2}_{kj}\ast {S}_j\right)}_{k-\mathrm{mer}}=\sum \limits_{j=1}^{N_{\mathrm{signalTrack}}}\sum \limits_{k=4}^{12}{\left(w{1}_{kj}\ast {\Delta }_j\right)}_{k-\mathrm{mer}}+\sum \limits_{j=1}^{N_{\mathrm{signalTrack}}}\sum \limits_{k=4}^{12}{\left(w{2}_{kj}\ast {S}_j\right)}_{k-\mathrm{mer}}=\mathrm{WS}\left(\Delta  \right)+\mathrm{WS}(S) $$

where *w*1_*kj*_ and *w*2_*kj*_ are the optimal weights learned from the training set of the eQTL model.

### Mouse transgenic reporter assays

Human enhancer regions (see Additional file 2: Tables S9-S10 for sequences) were PCR amplified from human genomic DNA (wild-type) or chemically synthesized by Integrated DNA Technologies (IDT) (5% top deM and 5% random non-deM mutations) and cloned into an Hsp68-promoter-LacZ reporter vector [46] using Gibson (New England Biolabs [NEB]) cloning [47]. Transgenic mouse embryos were generated by pronuclear injection, and F_0_ embryos were collected at E11.5 and stained for LacZ activity as previously described [45, 46]. The procedures for generating transgenic and engineered mice were reviewed and approved by the Lawrence Berkeley National Laboratory (LBNL) Animal Welfare and Research Committee.

## Additional files

Additional file 1: Supplemental information and supplementary figures. (DOCX 5769 kb)

Additional file 2: Supplementary tables. (XLSX 37 kb)

Additional file 3: Review history. (DOCX 396 kb)

## Data Availability

The GRCh37 (hg19) assembly of the human genome used in this study was downloaded from the UCSC Genome Browser [48, 49]. For the HepG2 and left ventricle enhancer set, we used the predicted active enhancers (state 8 and 9) from the expanded 18-state model of the Roadmap Epigenomics Project [32]. Promoter regions were defined as 1500 base pairs (bps) upstream and 500 bps downstream from a TSS and were excluded from the set of enhancers. The ChIP-seq peaks (narrowPeak format) of TFs and histone marks of HepG2 cell line were downloaded from the USCS Genome Browser ([50]; http://genome.ucsc.edu/). The multiz46way multiple sequence alignment of hg19 to other species were downloaded from UCSC genome browser [50]. The H3K27ac ChIP-seq peaks of liver tissues in mouse, cow, dog, and opossum originated from [51] were downloaded from EMBL-EBI ArrayExpress (accession number: E-MTAB-2633). The Hi-C contact map with 5-kb resolution in GM12878, HMEC, HUVEC, K562, and NHEK generated by [52] were downloaded from NCBI Gene Expression Omnibus (GEO) (accession number: GSE63525). Genetic variation data were downloaded from the 1000 Genomes Project [53]. The Sharpr-MPRAs (Massively Parallel Report Assays) regulatory scores were obtained from the study [54]. The gene expression profiles across 53 tissues were obtained from GTEx portal (GTEx Analysis V7 release) on May 9, 2019. For supplementary material, please refer to Additional files.

## Abbreviations

3D: Three dimensional
CAPE: CellulAr-dePendent dEactivating mutations
ChIP-seq: Chromatin immunoprecipitation experiments followed by sequencing
deMPs: Deactivating mutation positions
deMs: Deactivating mutations
DHSs: DNase I hypersensitive sites
Encode: Encyclopedia of DNA Elements
eQTLs: Expression quantitative trait loci
GO: Gene Ontology
GWAS: Genome-Wide Association Study
HepG2: Hepatocellular carcinoma
SNPs: Single-nucleotide polymorphisms
SVM: Support vector machine
TFBSs: Transcription factor binding sites
TFs: Transcription factors
TOR: Target of rapamycin
TSSs: Transcription start sites
