# Supplementary material for: Stable enhancers are active in development, and fragile enhancers are associated with evolutionary adaptation
Source: Genome Biol. 2019 Jul 15;20:140. doi: 10.1186/s13059-019-1750-z (PMC6631995; doi:10.1186/s13059-019-1750-z)
Supplement: Supplementary file 1 — Supplemental information and supplementary figures. (DOCX 5769 kb) [file 13059_2019_1750_MOESM1_ESM.docx]

**Supplementary Materials**

**Supplementary Information**

**Associating enhancer with their nearest gene**

The technology nowadays limited our knowledge of accurate identification of the target of enhancers, therefore, the 3D enhancer-promoter contact map is far from complete. The highest resolution of the Hi-C map for the majority of the cell lines is 5 kb [1]. This low resolution precludes us from identifying enhancer-promoter loops < 5 kb apart. Due to this limitation, in the original study of the published Hi-C data [1], only two loci ≥ 35 kb apart were examined for high-confident loops for 5-kb resolution, which results in the failure to identify contacts between a large portion of enhancers and their nearest TSSs. Since we do not have the high-resolution Hi-C data for the HepG2 cell line, we associate the enhancer with its nearest gene using GREAT [2] for functional annotation, considering that enhancers tend to interact with their nearest gene frequently enough that we would still be able to gain some useful information. First of all, we need to show that the functional annotation of these two sets of enhancers (fragile and stable) are very robust by using different rules of regulatory domain assignment in GREAT. Specifically, in addition to assigning an enhancer to its single nearest gene, we also apply the rules of “basal plus extension” and “two nearest gene” to associate enhancers with genes. The enrichment results show that the fragile enhancers are often enriched in the defense system and metabolism, whereas the stable enhancers are often associated with developmental system (Additional File 1: Fig. S15).

In spite of the limitation of the current technology and unavailability of Hi-C data in HepG2 cell line, to get a general idea on the proportion of enhancer linked to its nearest promoter, we took advantage of the Hi-C maps from the five cell lines which have 5 kb resolution: GM12878, HMEC, HUVEC, K562, and NHEK. Although we are unable to capture the Hi-C links between enhancers and their nearest TSS (<5 kb apart) due to technological limitations, around 50-60% of enhancers target to their nearest promoters (Additional File 1: Fig. S16A). On average, 55% of enhancers target to their nearest gene. Therefore, to offset the bias caused by assigning all enhancers to their nearest gene, we randomly relocated 45% of enhancers for each of the two sets of enhancers (fragile and stable) and ran GREAT to identify enriched functional groups of genes, and repeated this process for 10 times. Again, the enrichment results are very robust—the fragile enhancers are enriched in the metabolism and defense system, in contrast, the stable enhancers are enriched in the developmental system and regulation of transcription factor activity (Additional File 1: Fig. S16B).

Finally, the frequency of the cases where an enhancer target to a promoter no less than 1Mb away (such as the shh enhancer) should be very small. Specifically, only 0~4% of enhancers loop to the gene this afar (Additional File 1: Fig. S16A).

All the results above indicate that using GREAT with the single nearest gene rule to annotate the two sets of enhancers could provide fairly confident and robust functional enrichments in spite of the lack of an accurate 3D map of the genome.

**The cutoff of percentage of enhancers to define fragile/stable enhancers**

The reason we use 20% to define the three sets of enhancers is because: 1) we are interested in the outliers of enhancers that are either mostly enriched with deactivating mutations or depleted with deactivating mutations; and, 2) although there is often no clear cutoff in biology, we still need a cutoff that is sufficient to identify these outliers, similar to the definition of super enhancers. To show the overall trend of the correlation between functional conservation and densities of deactivating mutations in enhancer regions, we used a 10% percentile window to partition all the enhancers to 10 bins based on their deMP densities in order to compare their functional conservation levels by mapping all 10 bins to mouse liver enhancers. Accordant with our expectation, the level of functional conservation is generally negatively correlated with deMP density, with the top 20% and bottom 20% of enhancers showing the most striking difference on the level of functional conservation, whereas the middle 60% of enhancers showing somewhat similar extents of functional conservation (Additional File 1: Fig. S17).

**Comparison of enhancer activity**

We compared the regulatory activity of the three classes of enhancers using several datasets available for the human genome: H3K27ac and H3K4me1 ChIP-seq signal intensity [3], liver-specific TF ChIP-seq signal intensity [4, 5], and Sharpr-MPRA (Massively Parallel Report Assay) enhancer activity score [6].

We followed the procedure developed by the authors of Sharpr-MPRA to quantify the regulatory activity of a region using MaxPos Sharpr-MPRA score [6]. For this analysis, we used MaxPos Sharpr-MPRA region overlapping by at least 80% with the middle [-200bp, 200bp] window of HepG2 enhancers, and used the MaxPos scores of these Sharpr-MPRA sequences to quantify the regulatory activity of the three sets of HepG2 enhancers (Additional File 1: Fig. S12).

All the analysis of signal intensities of enhancer-specific histone marks (H3K27ac and H3K4me1) and liver-specific transcription factors, and the Sharpr-MPRA scores indicate that stable enhancers exhibit stronger enhancer activity compared to fragile enhancers, thus validating the hypothesis suggested by the Reviewer (Additional File 1: Fig. S12).

**Examining regulatory codes of three classes of enhancers using ChIP-seq peaks**

We leveraged all available 48 TF ChIP-seq datasets available for the HepG2 cell line. We analyzed TF-binding patterns in the three classes of HepG2 enhancers (stable, regular, and fragile enhancers; five-fold DHS regions were used as controls). The number of ChIP-seq peaks of a particular TF in a set of sequences was normalized by the total length of sequences in a dataset (Additional File 1: Fig. S6).

Consistent with the enrichment of *in silico* predicted TF motifs (Figure 4A), the stable enhancers are enriched for ChIP-seq peaks of FOXA1, FOXA2 and AP-1 (subunits: c-JUN, FOSL2), whereas the fragile enhancers are more enriched with binding of nuclear receptor factors HNF4A, HNF4G, and NR2F2 (Additional File 1: Fig. S6). This validates our reported differential enrichment of TFs in stable and fragile enhancers and confirms that the classes of TFs identified using an *in silico* approach are supported by the in vivo data analysis.

Next, to compare the TF-TF interaction patterns in the two classes of enhancers using ChIP-seq data, we first defined homotypic TF binding clusters (dubbed clusters of homotypic TF binding regions or clusters of homotypic TFBRs) as chromosomal segments that contain at least three TF ChIP-seq peaks within a 1kb window. The same three TF ChIP-seq peaks per 1kb definition was applied to clusters of different TFs to define heterotypic clusters of TFs. Due to the lack of single nucleotide resolution of TF ChIP-seq data (TF ChIP-seq peaks commonly span several hundred bps and contain dozens of TFBS motifs), a single TF ChIP-seq peak could very well contain a cluster (homotypic or heterotypic) of TFBS motifs and, thus, is not an ideal way for detecting homotypic TFBS binding. As a consequence, only a tiny portion of enhancers harbor clusters of homotypic TFBRs (Additional File 1: Fig. S18A). Therefore, the presented data analysis could be interpreted only as a conceptual validation of the *in silico* clustering results, but not a quantitative comparison. Despite these limitations, the fold-enrichment analysis shows that the homotypic ChIP-seq TFBRs are indeed enriched in stable enhancers, while heterotypic TFBRs are enriched in fragile enhancers (Additional File 1: Fig. S18B; regular enhancers used as a background for fold-enrichment computations).

Finally, to accurately quantify the TF-TF cooperativity pattern and capture the clusters of homotypic TFBRs supported by ChIP-seq data, we identified in silico TFBS clusters (homotypic and heterotypic) using only in silico TFBS sites residing within their corresponding TF ChIP-seq peaks. For example, we would keep an HNF4a predicted TFBS for this analysis only if it overlaps an HNF4a ChIP-seq peak. Our analysis shows that the stable enhancers are almost exclusively enriched for homotypic TFBS clusters, whereas heterotypic TFBS clusters dominate fragile enhancers (Figure 4D). This trend is consistent to the cooperativity patterns based on all potential TFBSs (Figure 4C) and further strengthens the conclusions of this study.

**Classifying TFs by tissue-biased patterns of expression**

We evaluated the tissue-specificity of the enriched TFs of the two sets of enhancers using the metric, $\tau$ [7], which ranges from 0 (consistent expression across all tissues) to 1 (expression limited to a single tissue/cell line). Gene expression profiles across 53 tissues were obtained from the GTEx portal (GTEx Analysis V7 release) on 05/09/2019. Consistent with the evolutionary resilience of ubiquitously-expressed genes in the mammalian liver [8], the TFs enriched in the stable enhancers exhibit a lower level of tissue-specificity compared to those enriched in fragile enhancers (Additional File 1: Fig. S13).

**Supplementary Figures**

Fig. S1

B

A

Characteristics of three sets of enhancers. A) Distribution of length of non-repetitive enhancer regions for the three sets of enhancers. P-values are calculated using Mann–Whitney–Wilcoxon test. B) Boxplot showing the densities of deMPs in the three sets of enhancers

Fig. S2

Fraction of genomic positions holding a SNP

deMPs are more conserved than non-repetitive enhancer positions according to comparison of the fraction of nucleotides carrying a SNP. The fraction above each bar shows the number of deMPs carrying SNP (numerator) against the number of non-repetitive enhancer positions carrying SNP (denominator). P-values are calculated using Mann–Whitney–Wilcoxon test.

Fig. S3


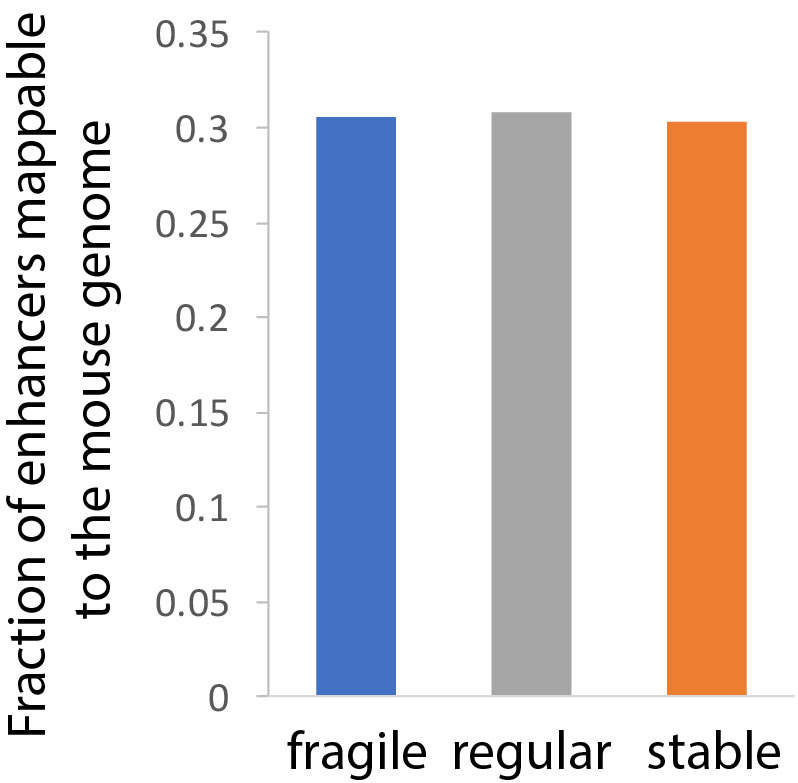


Fractions of enhancers that have orthoglous counterparts in the mouse genome.

Fig S4.

More orthologous sequences of stable enhancers in the four mammals have H3K27ac peaks than those of fragile enhancers. The p-value was calculated based on Fisher’s exact test. The species were selected as a representative of their corresponding placental order [9]. Orthologous regions were extracted based on multiz46way multiple sequence alignment of hg19 to other species.

Fig. S5

Comparisons of the two sets of enhancers with similar conservation levels of their fraction of functional conservation with mouse. The enhancers were partitioned based on their sequence conservation—the fraction of non-repetitive regions located within 46-way Plancental phastCon conserved elements. Fifty enhancers were sampled from each conservation bin for both classes and aligned to mouse; the process was repeated for 10 times. P-values were calculated based on Mann–Whitney U test. ** corresponds to p-value <= 0.005, * corresponds to p-value <= 0.01.

Fig. S6


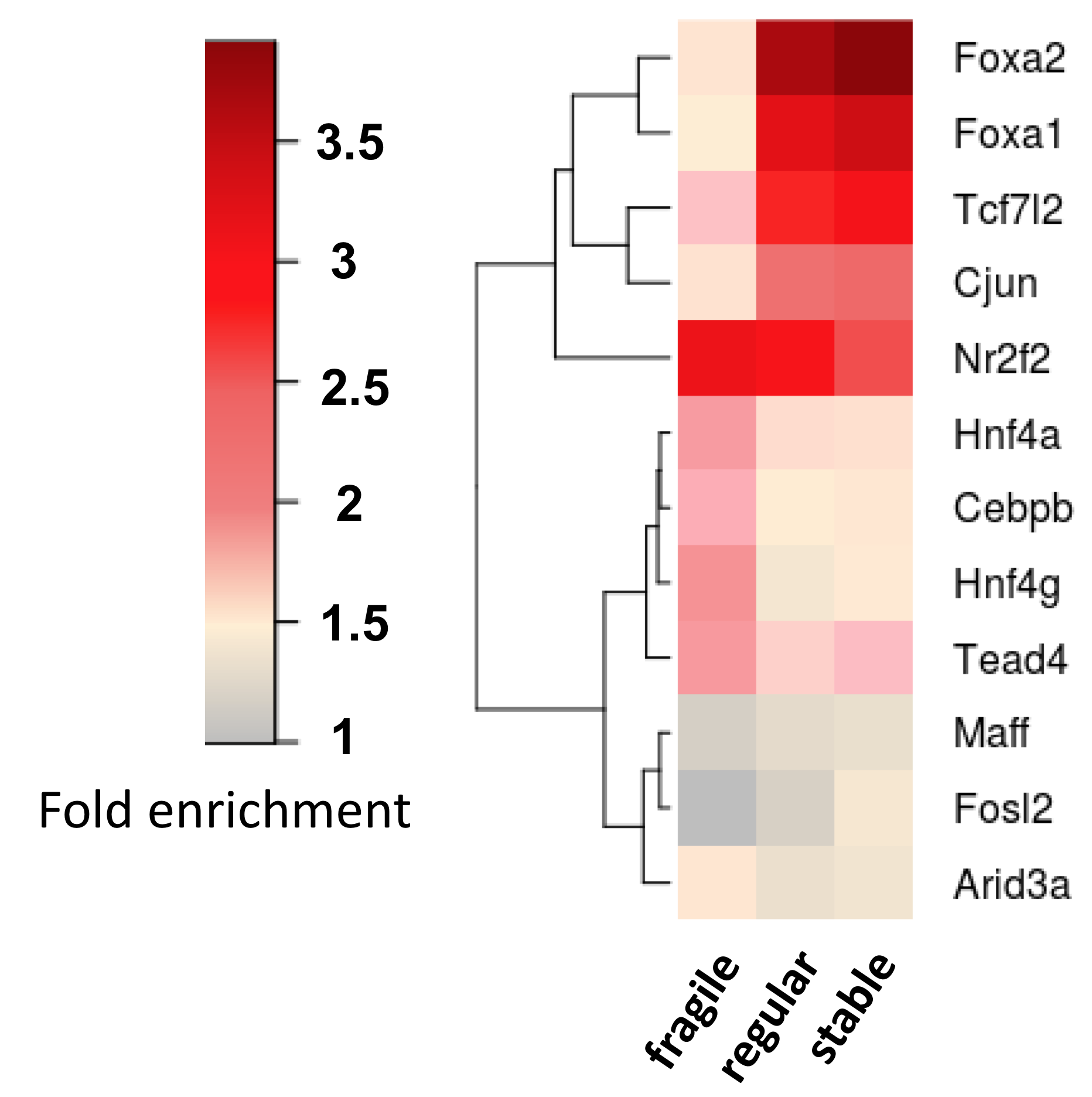


Different cohorts of TFBSs are enriched in enhancers with different densities of deMPs. The legend shows the range of fold enrichment of TFBSs in a set of enhancers relative to their negative control (random DHS region). Only the TFs with >= 1.2 of fold enrichment in at least one class of enhancer were included.

Fig. S7

The three sets of enhancers have similar densities of the enriched TFBSs. Only the TFBSs shown in Figure 4A were included in the analysis.

Fig. S8

Homotypic TFBS clusters are depleted of holding deMPs compared to other TF binding sites. A) Fraction of TFBSs contain at least one deMP. P-values were calculated based on Fisher’s exact test. B) Density of deMP (number of deMPs per base pair) of a region. ** refers to Binomial P-values <= 1.75e-55. Only enriched liver-specific TFs (TFBSs shown in Figure 4A) were included in the analysis.

Fig. S9


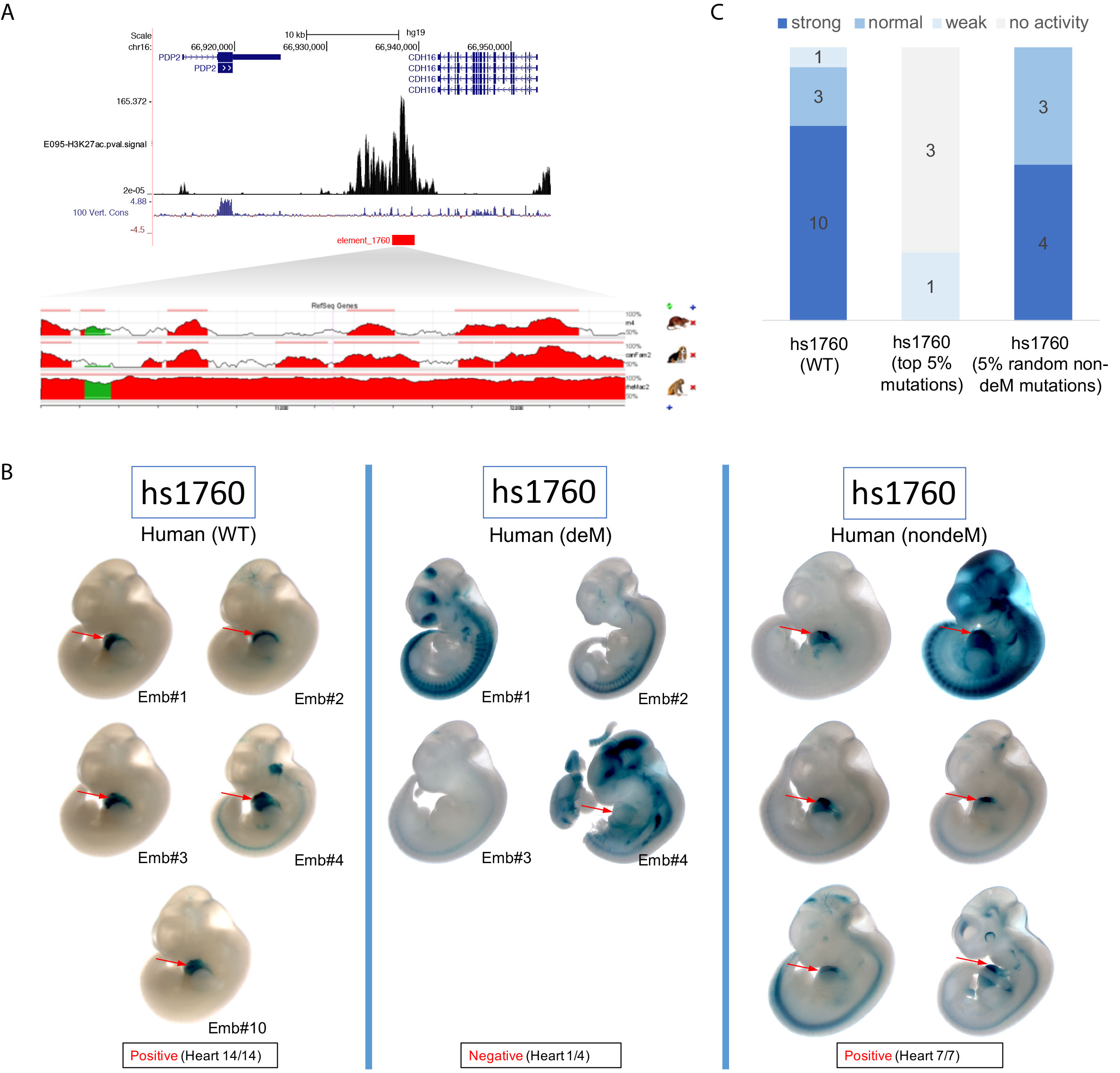


**CAPE accurately predicts change in LacZ expression in targeted mutagenesis of hs1760 in mouse embryonic heart (E11.5). Schematic of the human hs1760 heart enhancer locus is shown on the top. Three versions of hs1760: hs1760 wild type (WT), hs1760 with top 5% mutations (deM), and hs1760 with 5% random mutations (non-deM) are shown.** A) The H3K27ac signal and enrichment of ECRs of hs1760. B) Both the wild type enhancer sequence and 5% non-deM random mutations could drive LacZ expression of hs1760 in E11.5 mouse heart; by contrast, the top 5% mutations including deMs could deactivate this enhancer in E11.5 mouse heart. C) Expression profile of the three versions of hs1760 in E11.5 mouse heart based on the four categories of expression levels. The lacZ staining in the heart was highlighted with a red arrow. The hg19 genomic coordinates were obtained from UCSC genome browser (<http://genome.ucsc.edu/>). The H3K27ac enrichment data of human heart left ventricle was obtained from ROADMAP epigenomics project [3]. The overlapping of hs1760 with ECR regions was obtained from ECR browser (<https://ecrbrowser.dcode.org/>). The images of the wildtype hs1760 were obtained from VISTA Enhancer Browser (<https://enhancer.lbl.gov/>), only representative embryos are shown for hs1760 WT.

Fig. S10


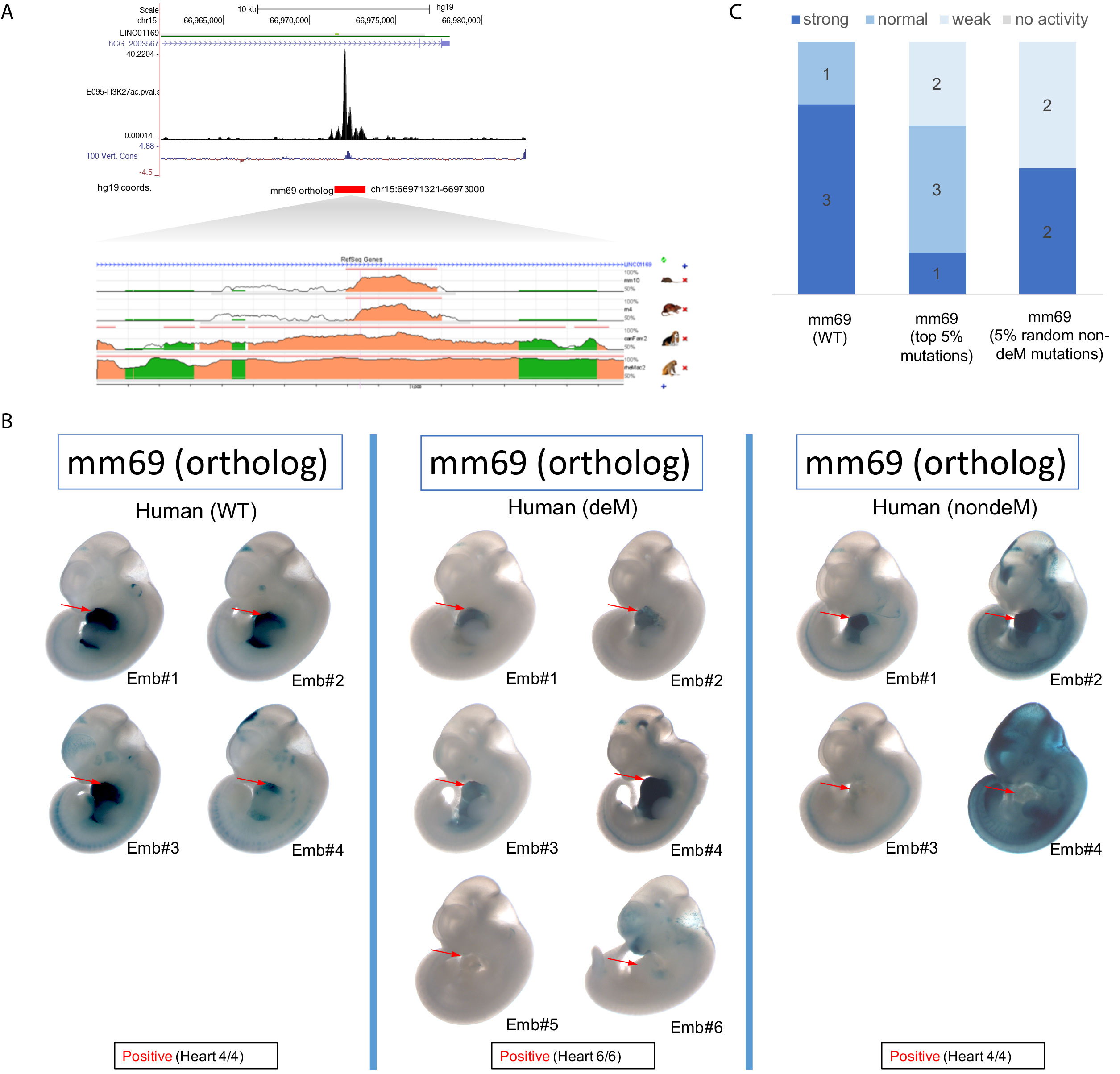


**CAPE accurately predicts change in LacZ expression in targeted mutagenesis of human ortholog of mm69 in mouse embryonic heart (E11.5). Three versions of mm69 (ortholog): mm69 (ortholog) wild type (WT), mm69 (ortholog) with top 5% mutations (deM), and mm69 (ortholog) with 5% random mutations (non-deM) are shown.** A) The H3K27ac signal and enrichment of ECRs of mm69 ortholog. B) Both the wild type enhancer sequence and 5% non-deM random mutations could drive LacZ expression of mm69 ortholog in E11.5 mouse heart; by contrast, the top 5% mutations including deMs could diminish the activity of this enhancer in E11.5 mouse heart. C) Expression profile of the three versions of mm69 ortholog in E11.5 mouse heart based on the four categories of expression levels. The lacZ staining in the heart was highlighted with a red arrow. The hg19 genomic coordinates were obtained from UCSC genome browser (<http://genome.ucsc.edu/>). The H3K27ac enrichment data of human heart left ventricle was obtained from ROADMAP epigenomics project [3]. The overlapping of mm69 ortholog with ECR regions was obtained from ECR browser (<https://ecrbrowser.dcode.org/>).

Fig. S11

Gene loci associated with fragile enhancers have length distributions similar to those associated with stable enhancers.

Fig. S12

Stronger enhancer activity in stable enhancers. A) Stable enhancers show higher H3K27ac signal intensity. For enhancers with multiple H3K27ac peaks, only the peaks located closest to the enhancer center were included. B) Stable enhancers have greater Sharpr-MPRA regulatory scores. The number within parentheses below each boxplot gives the number of elements examined in Sharpr-MPRAs. C) Stable enhancers show stronger H3K4me1 signal intensity. D) Stable enhancers exhibit stronger TF binding ChIP-seq signal. P-values were calculated based on Mann–Whitney U test. * refers to P-value < 1e-3, ** refers to P-value < 1e-5.

Fig. S13


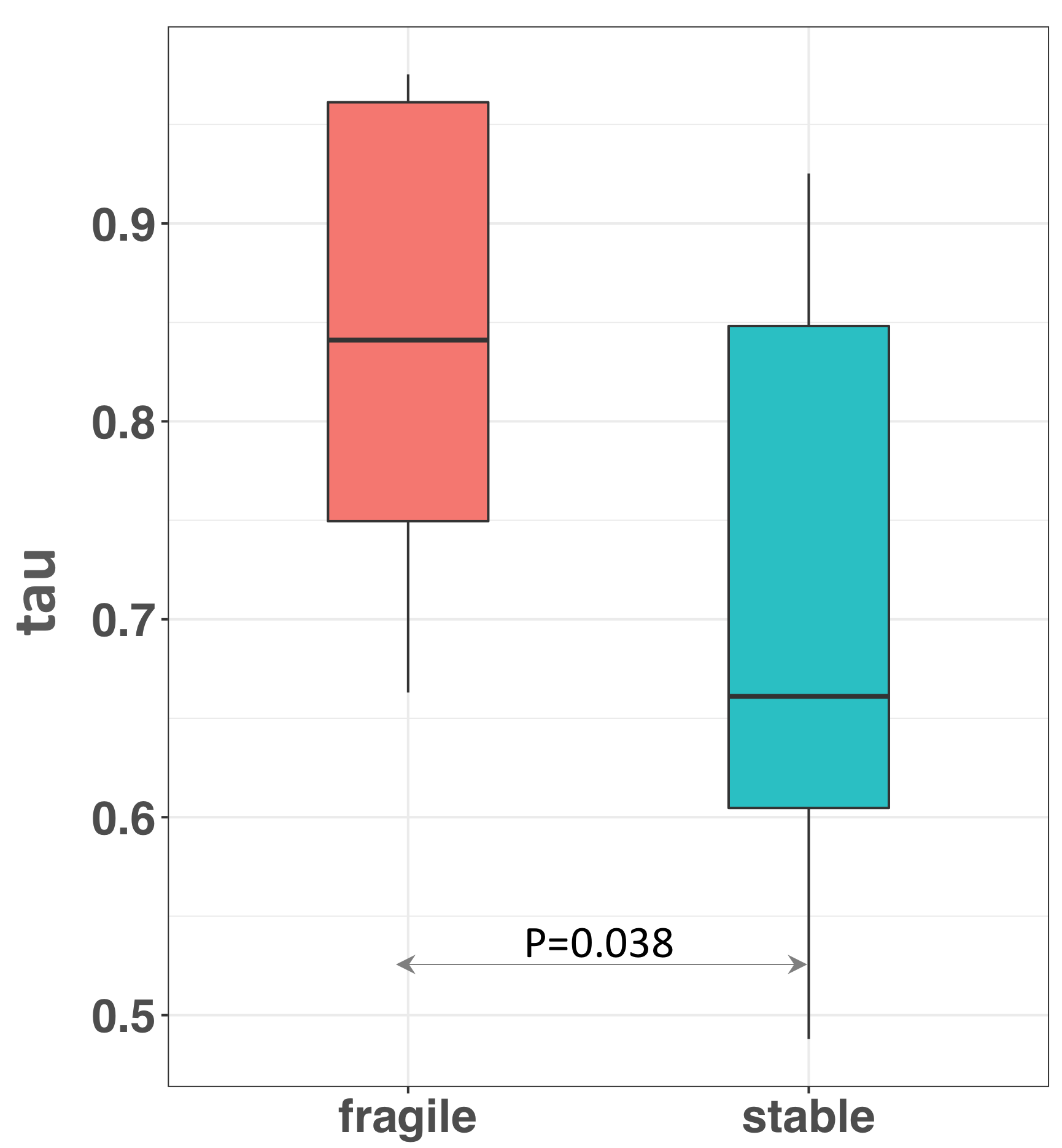


Tissue-specificity (τ) of the TFs enriched in fragile enhancers (NR1I2, HNF4A, NR1H4, PPARG, NR4A1, PPARA, NR2F1, NR2C2, and NR2F6), and those enriched in stable enhancers (FOXA2, FOXA1, FOXD1, FOXO4, FOXO3, JUNB (AP-1), JUND (AP-1), FOSL2 (AP-1), and TCF7L2). The p-values were calculated based on Mann–Whitney U test.

Fig. S14


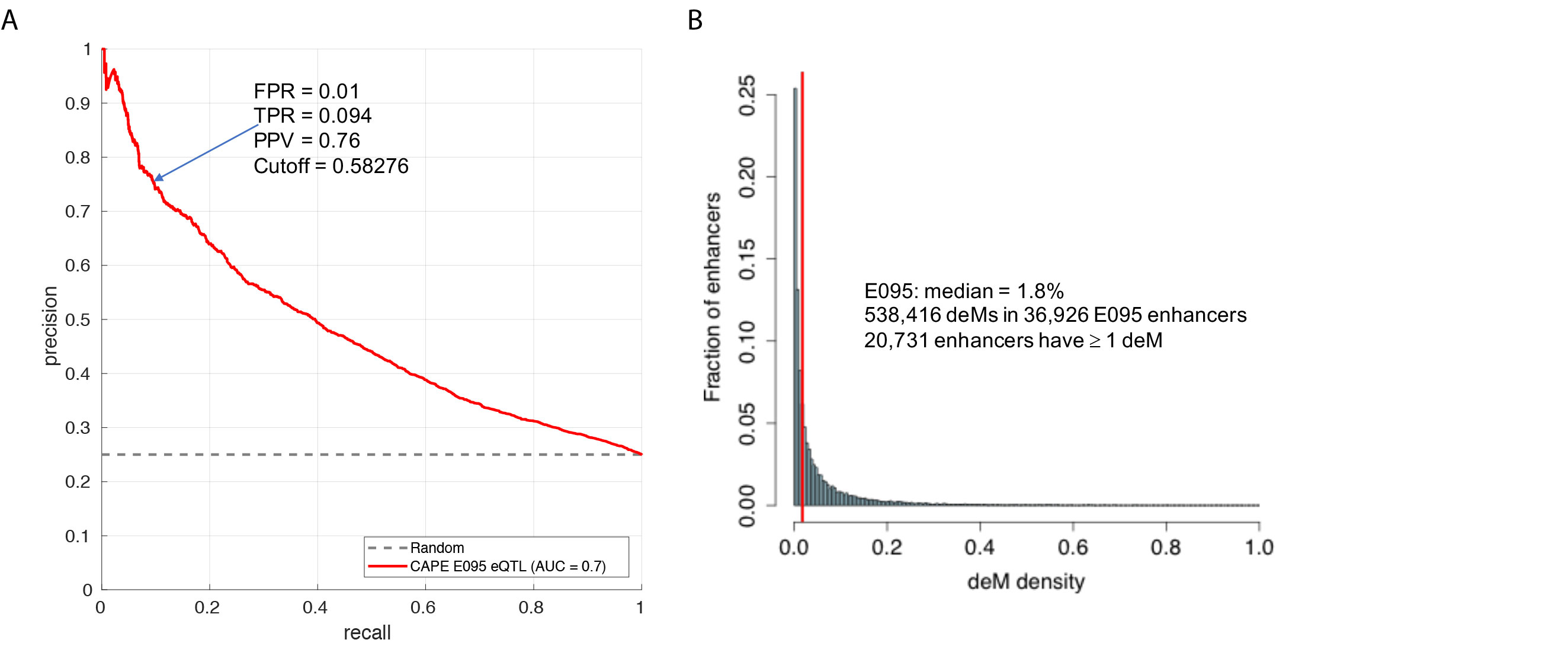


Applying CAPE to identify deMPs in left ventricle. A) CAPE was trained on human left ventricle eQTLs. The mutations with CAPE score ≥ 0.58276 (FPR ≤ 0.01) were considered as deMs. B) Distribution of densities of deMPs in left ventricle (E095) enhancers. The top, middle, bottom 20% of enhancers correspond to fragile, regular, and stable enhancers, respectively.

Fig. S15


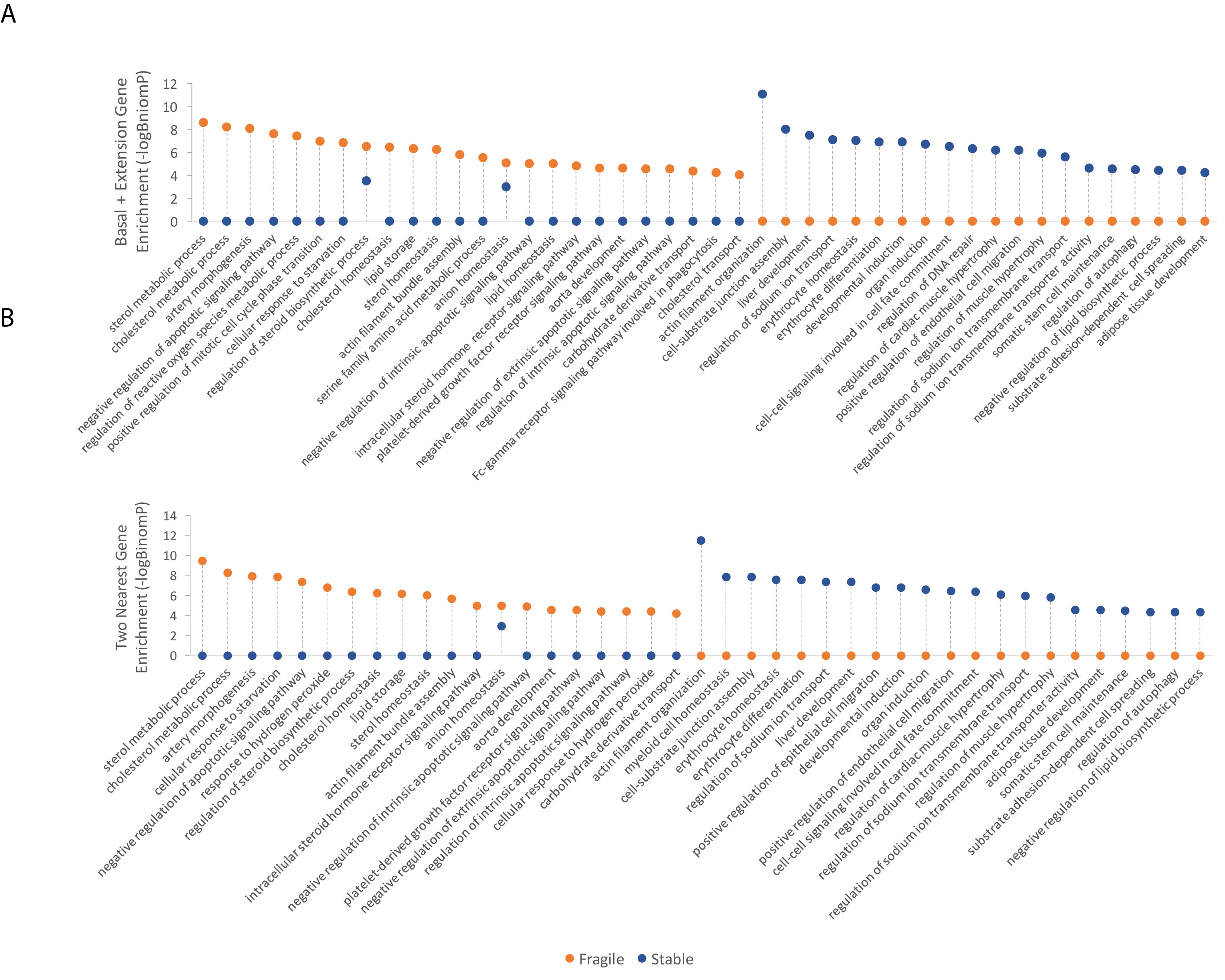


GO enrichment using GREAT with the other two methods to associate enhancer to genes. A) Basal plus extension. B) Two nearest genes.

Fig. S16


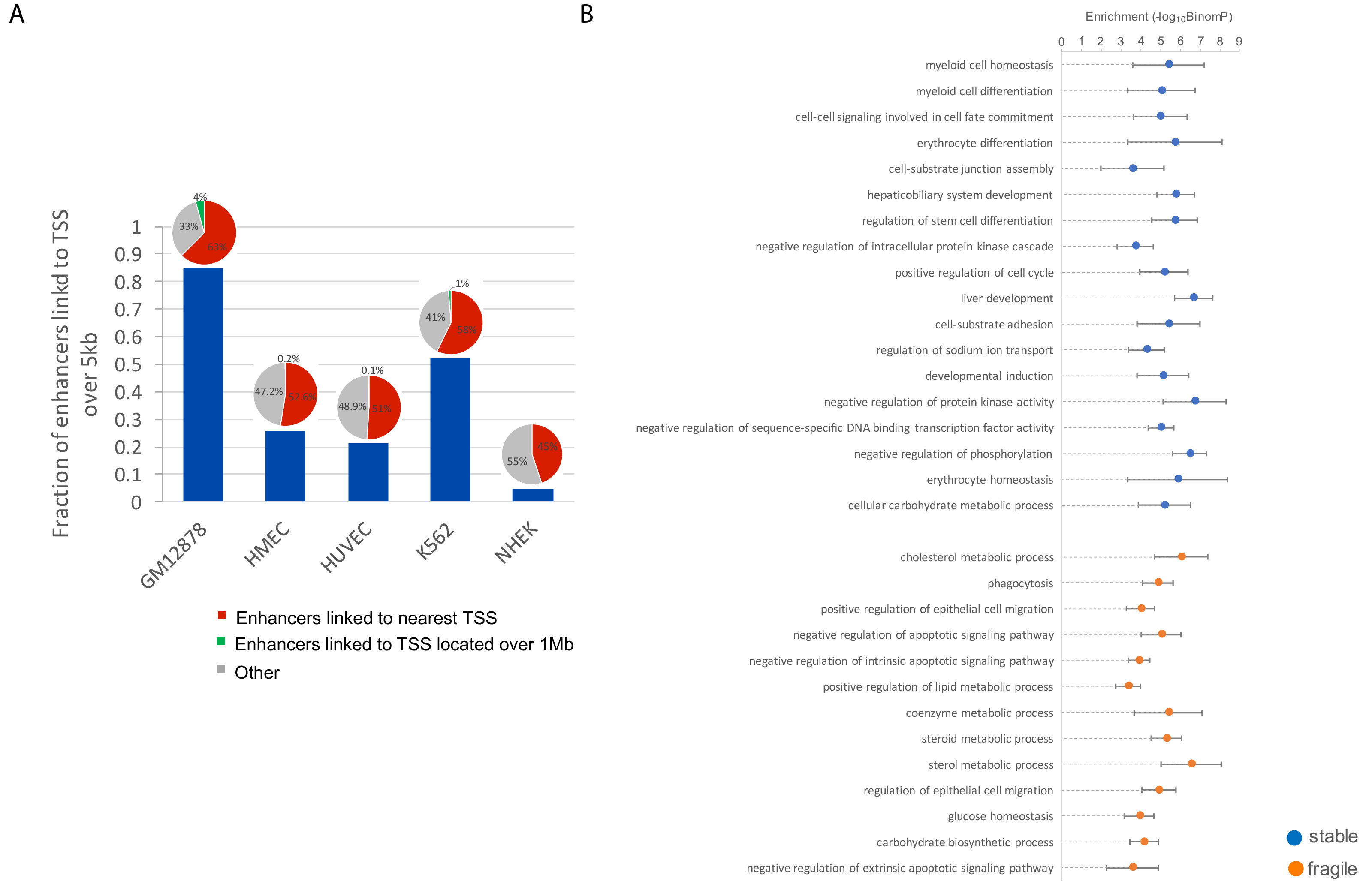


A) Fraction of enhancers linked to nearest promoters compared to fraction of enhancers linked to TSS no less than 1Mb away based on Hi-C data. B) Gene annotation enrichment results of GREAT for the stable and fragile enhancers with 45% randomly relocated for 10 times. The dots represent the mean value of the enrichment, and the error bars show the standard deviation. Only the GO terms enriched for at least five times are included.

Fig. S17

Correlation between deMP density and the extent of preservation of regulatory activity of all HepG2 enhancers. Enhancers were sorted decreasingly by their densities of deMP and binned to 10 percentiles.

Fig. S18


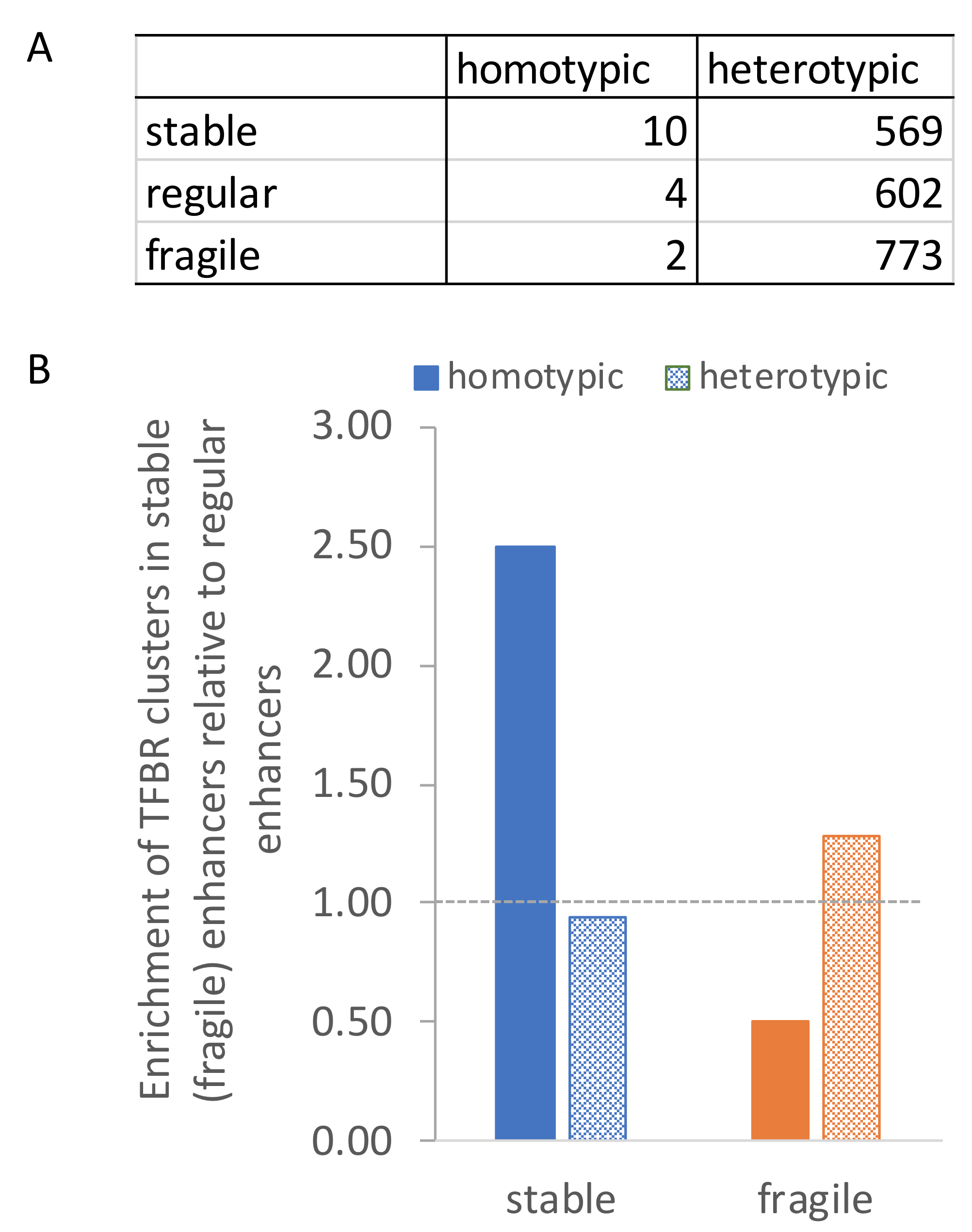


Cooperativity of TFs using ChIP-seq peaks. A) Number of clusters of homotypic/heterotypic TF binding regions (TFBRs) in the three classes of enhancers. B) Enrichment of TFBR clusters in stable (or fragile) enhancers relative to regular enhancers. The dashed line represents the background where fold enrichment = 1.

References

1. Rao SS, Huntley MH, Durand NC, Stamenova EK, Bochkov ID, Robinson JT, Sanborn AL, Machol I, Omer AD, Lander ES, Aiden EL: **A 3D map of the human genome at kilobase resolution reveals principles of chromatin looping.** *Cell* 2014, **159:**1665-1680.

2. McLean CY, Bristor D, Hiller M, Clarke SL, Schaar BT, Lowe CB, Wenger AM, Bejerano G: **GREAT improves functional interpretation of cis-regulatory regions.** *Nat Biotechnol* 2010, **28:**495-501.

3. Kundaje A, Meuleman W, Ernst J, Bilenky M, Yen A, Heravi-Moussavi A, Kheradpour P, Zhang Z, Wang J, Ziller MJ, et al: **Integrative analysis of 111 reference human epigenomes.** *Nature* 2015, **518:**317-330.

4. Bernstein BE, Birney E, Dunham I, Green ED, Gunter C, Snyder M: **An integrated encyclopedia of DNA elements in the human genome.** *Nature* 2012, **489:**57-74.

5. Gertz J, Savic D, Varley KE, Partridge EC, Safi A, Jain P, Cooper GM, Reddy TE, Crawford GE, Myers RM: **Distinct properties of cell-type-specific and shared transcription factor binding sites.** *Mol Cell* 2013, **52:**25-36.

6. Ernst J, Melnikov A, Zhang X, Wang L, Rogov P, Mikkelsen TS, Kellis M: **Genome-scale high-resolution mapping of activating and repressive nucleotides in regulatory regions.** *Nat Biotechnol* 2016, **34:**1180-1190.

7. Yanai I, Benjamin H, Shmoish M, Chalifa-Caspi V, Shklar M, Ophir R, Bar-Even A, Horn-Saban S, Safran M, Domany E, et al: **Genome-wide midrange transcription profiles reveal expression level relationships in human tissue specification.** *Bioinformatics* 2005, **21:**650-659.

8. Berthelot C, Villar D, Horvath JE, Odom DT, Flicek P: **Complexity and conservation of regulatory landscapes underlie evolutionary resilience of mammalian gene expression.** *Nat Ecol Evol* 2018, **2:**152-163.

9. Villar D, Berthelot C, Aldridge S, Rayner TF, Lukk M, Pignatelli M, Park TJ, Deaville R, Erichsen JT, Jasinska AJ, et al: **Enhancer evolution across 20 mammalian species.** *Cell* 2015, **160:**554-566.
