# Supplementary material for: Stable enhancers are active in development, and fragile enhancers are associated with evolutionary adaptation
Source: Genome Biol. 2019 Jul 15;20:140. doi: 10.1186/s13059-019-1750-z (PMC6631995; doi:10.1186/s13059-019-1750-z)
Supplement: Supplementary file 3 — Review history. (DOCX 396 kb) [file 13059_2019_1750_MOESM3_ESM.docx]

**First Round of Review**

**Reviewer 1**

This manuscript by Li et al. applies their recently developed classifier (CAPE) to identify variants that could disturb transcription factor (TF) binding in HepG2 enhancers, which they term deactivating mutations (deMs). The authors show that HepG2 enhancers contain a wide range of putative deM positions (deMPs) and thus proceed to classify them into stable, regular and fragile enhancers based on their deMP density. They then show that stable enhancers (containing the lowest number of putative deMPs) are characterized by conserved activity across mammals, are proximal to developmental genes and primarily bound by pioneer TFs, such as those of the FOX family, often in a homotypic fashion (i.e., multiple binding sites of the same TF occur in the same regulatory region). On the other hand, fragile enhancers show less conserved activity, are enriched near immune response and metabolic genes, primarily bound by nuclear receptors and characterized by heterotypic binding. By decomposing their classifier, the authors show that enhancer fragility is primarily determined by the adjacent TF binding sites and the larger chromatin context rather than the cognate motif itself. Then, they proceed to validate their deM predictions by performing targeted mutagenesis followed by a lacZ reported assay on two human heart enhancers.

This work provides important insights into how sequence variation can impact transcription factor binding and ultimately enhancer activity. Importantly, it highlights the significance of the local sequence and chromatin context in buffering the effect of single nucleotide variation in transcription factor binding. The authors succeed in concisely reporting that their observations correspond to associations and not causal relationships through the largest part of the manuscript; however, this is not the case with their title, and the conclusions of their abstract and discussion sections (see below). 

Major comments
1. Although for most of their manuscripts the authors clearly state that their reported results are associations, their title ("Two classes of enhancer stability: stable enhancers active in development and fragile enhancers contributing to evolutionary adaptation"), abstract (P2 L23 "This dichotomy of enhancer activity […] argues for a bimodal evolution of gene regulatory programs in vertebrates with specifically encoded stable enhancers establishing an evolutionary reliable foundation of development and differently encoded fragile enhancers contributing to the adaptation of species", and discussion (P15 L 35-42) suggest that the two different classes of enhancers with respect to their deM density are responsible/ contribute to the different evolutionary fates between the two classes. Although this is to some extent possible, it is not shown by the analyses provided by the authors. An alternative interpretation of their results could be the following: developmental enhancers show high activity conservation (i.e., due to developmental constraints) and are enriched for pioneer TF binding, which primarily occurs in homotypic fashion. Due to the multiple occurrences of motifs of the same TF, single nucleotide changes are less likely to impact TF binding or enhancer activity. The same logic applies to fragile enhancers. The crucial difference with the interpretation provided by the authors is that differences in deM density could be a mere consequence of the different properties between developmental enhancers and those regulating metabolic or immune genes, rather than the cause that drives the other observations.

2. The authors claim that stable and fragile enhancers are characterized by a similar degree of sequence constraint (P7 L7: "… although the three sets of enhancers share similar evolutionary constraint on the sequence level, the high density of deMPs make the fragile enhancer more prone to single-nucleotide deactivating mutations and, therefore, harder to maintain its regulatory function during evolution"). However, their phastCons analysis (Figure 2D) clearly shows that stable enhancers are more conserved at the sequence level. Given the fact that sequence and activity conservation of enhancers are known to correlate (for example see Villar et al. Cell 2015), this could be an important confounder in the reported results. The authors should either discuss the possibility that the increased enhancer activity conservation of stable enhancers is associated with their stronger sequence conservation (rather than the frequency of deMPs) or show that their observations hold if they subsample enhancers of similar sequence constraint between the two classes.

3. P8 L28: "In addition, it has been observed that a significant number of genes involved in cancer predate the origin of multicellularity, which is consistent with the strong conservation of stable enhancers". This is quite a severe overstatement: The fact that cancer genes are conserved across eukaryotes cannot be extrapolated to the conservation of their enhancers (see also Emera et al. PNAS 2016: genes with ancient functions are associated with mammal-specific enhancers in the neocortex). Consistently, many of the cellular maintenance genes the authors associate with fragile enhancers are also very ancient, but this doesn't seem to be the case for their enhancers.

4. The authors interpret the weak effects of introducing deMs to their second construct (human ortholog of mm69) as likely associated "with the enrichment of homotypic TFBS clusters in this enhancer" (P12 L40). However, if I understand the CAPE method correctly, adjacent TF binding and chromatin context is already taken into consideration when determining deMs over non-deM mutations. Thus, this doesn't seem like a probable explanation for the lack of striking differences between random and deMs in the same enhancer. No classifier is expected to give perfect predictions and functional assays have a considerable degree of biological variability, but the authors should be transparent about these limitations.

Minor comments
1. P4 L7: "we observed that the deMP densities are correlated with the functional and evolutionary stability of enhancers". I assume that the authors mean "negatively correlated" instead.
2. P13 L9: "Due to the high functional constraint on deMPs, fragile enhancers are prone to one single nucleotide deactivation and, therefore, evolve faster". The authors should specify that they refer to conservation of activity (rather than sequence). 
3. P14 L47: loose -> lose
4. Although the authors attempt to validate their deMs predictions in heart enhancers, all other conclusions are exclusively supported by observations in HepG2 cells. However, this is a single cancer cell line, only remotely reflecting normal development. Thus, it is unclear whether these results can be generalized to enhancers active in primary cells or during organ development. This needs to be transparently acknowledged by the authors.

**Reviewer 2**

The authors present an interesting analysis on the genomic properties of enhancer elements, by focusing on prediction of transcription factor binding deactivating mutations (deMs). By leveraging a range of published datasets in liver tissue and the human liver carcinoma cell line HepG2, the authors categorise enhancers into two main categories (stable and fragile) based on their predicted density of deMs. The authors' results indicate stable and fragile enhancers associate with different levels of evolutionary conservation (both in terms of sequence constraint and epigenomic conservation of histone marks) and distinct biological pathways. Prediction of transcription factor binding sites and their enrichment in each set of enhancers leads the authors to propose specific transcription factors and motif-binding configurations to be preferentially associated with each enhancer category. Lastly, the authors provide a proof-of-principle experimental validation by performing mouse reporter assays with two stable enhancer elements (active in heart tissue) and their mutated variants.

Overall, we found the manuscript reports an interesting analysis with potential broad implications for enhancer biology. Nevertheless, we feel the manuscript needs to be conceptually improved in several areas, specifically with additional analyses and discussion points.

Major comments:

- It is unclear from the presented results whether stable and fragile enhancers may associate with different levels of enhancer activity. Both ChIP-seq signal intensity and in vitro reporter activity can be used as proxy measurements of enhancer activity, and there are several published resources the authors could exploit to address this question (e.g. see (Kheradpour, Ernst et al., 2013)and (Stefflova, Thybert et al., 2013)). Are stable enhancers also associated with higher levels of enhancer activity compared to fragile enhancers?

- The authors analyse sequence properties of stable and fragile enhancers by focusing on in silico prediction of transcription factor binding sites. While this approach is informative, it obviates the known in vivo binding of transcription factors to a small sub-section of predicted binding sites. Moreover, the authors' results suggest different modes of transcription factor binding associate with stable and fragile enhancers. These results would be strengthened by an analysis of transcription factor cooperativity in the two sets of enhancers, which could for example leverage high-quality ChIP-seq data for liver-specific transcription factor binding in mouse liver (Stefflova et al., 2013).

Minor comments:

- It is not very clear from the results description in the main text whether the manuscript is solely focusing on distal enhancers, or whether the analysed regions also include proximal regulatory sequences. This is included in the supplemental methods but should be clarified in the main text, given their known differences in evolutionary conservation and stability. In this regard, the authors are focusing on HepG2 / liver enhancers and attributing an enrichment of liver-specific transcription factors to stable enhancers. This analysis is somewhat reductionist, and it may be useful to consider what is the observed tissue-specificity in the expression of the transcription factors the authors predict to be enriched in each set of enhancers. For instance, are stable enhancers also enriched for binding of ubiquitously expressed transcription factors, as could be expected from the evolutionary resilience of ubiquitously-expressed genes in the mammalian liver (Berthelot, Villar et al., 2018)?

- The concept of deMs, how they are predicted and what are the caveats in this computational approach should be clarified in the manuscript (i.e. to make it more accessible to non-specialists). This is a central concept in the study but is succinctly described in the main results section. It would also be useful to mention the background controls used in this method in the main results.

- The authors should consider citing the primary publications from which they are leveraging published ChIP-seq data. The datasets and accession numbers are given in the Methods section, but it would be useful to cite the relevant papers in the main manuscript for clarity.

- The authors may want to clarify / justify their focus on the two extreme sets of enhancers (stable and fragile), as the "regular" category is largely omitted in the Figures. This is somewhat covered in the supplemental info but would be useful to allude to in the main text.

- The format in some of the Figures needs to be revised:
- The scale in Figure 2D could be adjusted to the dispersion observed in the data. As presented, it obscures the differences in medians across the three sets of enhancers.
- Gene ontology terms in Figure 3A are unreadable. A vertical layout of this panel would aid readability
- The heatmap scale in Figure 4A could be annotated as fold change, again to help readability.
- The format and layout of Figure 5 should be improved (e.g. some of the axes labels are illegible).

- The discussion section could be improved in terms of balance and integration of the results with existing observations in the field:
- The discussion of tissue-specific transcription factor motif enrichment in stable enhancers (page 13, lines 47-54) somewhat obviates gene size as a potential confounder (i.e. transcription factor genes are larger than average, so more regulatory elements are associated to them and these may be larger on average). It is also unclear whether the authors analyses can correct for gene size.
- Many of the authors observations appear consistent with classic models of enhancer grammar (e.g. enhanceosome and billboard models). It would be helpful to include a discussion of these and related models (Spitz & Furlong, 2012) and how the observed properties of stable and fragile enhancers may relate to them. 

- Minor revision of English language is needed throughout the manuscript.

References:

Berthelot C, Villar D, Horvath JE, Odom DT, Flicek P (2018) Complexity and conservation of regulatory landscapes underlie evolutionary resilience of mammalian gene expression. Nature ecology & evolution 2: 152-163

Kheradpour P, Ernst J, Melnikov A, Rogov P, Wang L, Zhang X, Alston J, Mikkelsen TS, Kellis M (2013) Systematic dissection of regulatory motifs in 2000 predicted human enhancers using a massively parallel reporter assay. Genome research 23: 800-11

Spitz F, Furlong EE (2012) Transcription factors: from enhancer binding to developmental control. Nature reviews Genetics 13: 613-26

Stefflova K, Thybert D, Wilson MD, Streeter I, Aleksic J, Karagianni P, Brazma A, Adams DJ, Talianidis I, Marioni JC, Flicek P, Odom DT (2013) Cooperativity and rapid evolution of cobound transcription factors in closely related mammals. Cell 154: 530-40

**Authors’ response to reviewers**

**Reviewer #1:**

*This work provides important insights into how sequence variation can impact transcription*

*factor binding and ultimately enhancer activity. Importantly, it highlights the significance of the*

*local sequence and chromatin context in buffering the effect of single nucleotide variation in*

*transcription factor binding. The authors succeed in concisely reporting that their observations*

*correspond to associations and not causal relationships through the largest part of the*

*manuscript; however, this is not the case with their title, and the conclusions of their abstract*

*and discussion sections (see below).*

The reviewer is absolutely correct; we tried to separate associations from causality, and we

apologize for the instances where we failed to adhere to this standard. Our response below

details how we addressed this in the revised version of the manuscript.

*Major comments*

*1. Although for most of their manuscripts the authors clearly state that their reported results*

*are associations, their title ("Two classes of enhancer stability: stable enhancers active in*

*development and fragile enhancers contributing to evolutionary adaptation"), abstract (P2 L23*

*"This dichotomy of enhancer activity […] argues for a bimodal evolution of gene regulatory*

*programs in vertebrates with specifically encoded stable enhancers establishing an evolutionary*

*reliable foundation of development and differently encoded fragile enhancers contributing to*

*the adaptation of species", and discussion (P15 L 35-42) suggest that the two different classes*

*of enhancers with respect to their deM density are responsible/ contribute to the different*

*evolutionary fates between the two classes. Although this is to some extent possible, it is not*

*shown by the analyses provided by the authors. An alternative interpretation of their results*

*could be the following: developmental enhancers*

*show high activity conservation (i.e., due to developmental constraints) and are enriched for*

*pioneer TF binding, which primarily occurs in homotypic fashion. Due to the multiple*

*occurrences of motifs of the same TF, single nucleotide changes are less likely to impact TF*

*binding or enhancer activity. The same logic applies to fragile enhancers. The crucial difference*

*with the interpretation provided by the authors is that differences in deM density could be a*

*mere consequence of the different properties between developmental enhancers and those*

*regulating metabolic or immune genes, rather than the cause that drives the other*

*observations.*

We agree with the reviewer on this point that the two sets of enhancers are associated with

different biological processes with a different extent of evolutionary constraint instead of the

activity of these two enhancer sets being the driving force behind development and evolution.

The title has been changed to “*Two classes of enhancer stability: stable enhancers active in*

*development and fragile enhancers* ***associated*** *with evolutionary adaptation*”. The conclusions in the

abstract has been restated as “*This dichotomy of enhancer activity is conserved across different*

*tissues, has a specific footprint in epigenetic profiles, and argues for a bimodal evolution of gene*

*regulatory programs in vertebrates with specifically encoded stable enhancers* ***residing at the***

***evolutionary reliable foundation*** *of development and differently encoded fragile enhancers being*

***associated with*** *the adaptation of species.*” We also rephrased the corresponding section in

Discussion as “*In short, with the stable enhancers at the foundation for development and transcription factor activity and the fragile enhancers* ***linked to*** *an adaptable regulatory program for the defense system and cellular maintenance, this bimodal system* ***contributes*** *to the fitness and adaptation of species*.”

In addition, by performing an extra analysis on the deMP density we observed that

deMPs are depleted in homotypic TFBS clusters relative to other transcription factor binding

sites (Figure S8AB), which further shows the strong association between the enrichment of

homotypic TFBS clusters and the depletion of deMPs. More importantly, the density of deMPs

in the non-TFBS enhancer regions is significantly depleted as compared to the binding sites of

transcription factors (Figure S8B), suggesting that deMs primarily stem from the mutations

disrupting the binding of essential transcription factors and/or co-factors, whereas the

enrichment of homotypic TFBS clusters may buffer genetic perturbations that affect one of the

motifs. Therefore, the stable enhancers enriched with homotypic TFBS clusters are likely to be

depleted of deMPs compared to fragile enhancers. We have added the following statement to

the revised version of the manuscript (Page 11): “*On the other hand, homotypic TFBS clusters are*

*depleted of deMPs as compared to other TF binding sites, although deMPs tend to target the binding*

*sites of essential liver-specific transcription factors compared to other enhancer regions (Figure S8),*

*suggesting that deMs primarily stem from mutations disrupting the binding of essential transcription*

*factors or co-factors, whereas the enrichment of homotypic TFBS clusters may alleviate the impact of*

*single nucleotide changes on TF binding or enhancer activity. Therefore, the stable enhancers enriched with homotypic TFBS clusters are likely to be depleted of deMPs compared to fragile enhancers.*”

**A**	**B**

**Figure S8. Homotypic TFBS clusters are depleted of holding deMPs compared to other TF binding sites. A) Fraction of TFBSs contain at least one deMP. P-values were calculated based on Fisher’s exact test. B) Density of deMP (number of deMPs per base pair) of a region. ** refers to Binomial P-values <= 1.75e-55. Only enriched liver specific TFs (TFBSs shown in Figure 4A) were included in the analysis**

*2. The authors claim that stable and fragile enhancers are characterized by a similar degree*

*of sequence constraint (P7 L7: "… although the three sets of enhancers share similar*

*evolutionary constraint on the sequence level, the high density of deMPs make the fragile*

*enhancer more prone to single-nucleotide deactivating mutations and, therefore, harder to*

*maintain its regulatory function during evolution"). However, their phastCons analysis (Figure 2D) clearly shows that stable enhancers are more conserved at the sequence level. Given the fact that sequence and activity conservation of enhancers are known to correlate (for example see Villar et al. Cell 2015), this could be an important confounder in the reported results. The authors should either discuss the possibility that the increased enhancer activity conservation of stable enhancers is associated with their stronger sequence conservation (rather than the frequency of deMPs) or show that their observations hold if they subsample enhancers of similar sequence constraint between the two classes.*

Following the reviewer’s suggestion, we decoupled the impact of sequence conservation and multiplicity of deMPs on functional conservation by partitioning two classes of enhancers into five bins based on their degree of phastCons conservation. For each bin, we compared the level of functional conservation between the two classes of enhancers by subsampling enhancers between the two classes. Specifically, 50 enhancers were sampled from each conservation bin for both classes and aligned to mouse, and this analysis was repeated 10 times. We observed that for the enhancers at the same sequence conservation level, fragile enhancers are consistently less functionally conserved relative to stable enhancers. Given these new data supporting the hypothesis of the functional conservation of stable enhancers, we have restated the corresponding results as following (Page 7): “*Fragile enhancers display a significantly lower degree of sequence conservation as compared to stable enhancers (phastCons scores at non-repetitive DNA regions; Figure 2D). Fragile enhancers are also less conserved at the functional level (as measured by the fraction of the human/mouse enhancer orthologs that are active as enhancers in the same tissue in both species). To investigate whether the lower functional conservation of fragile enhancers is the consequence of the multiplicity of deMPs apart from their lower degree of sequence conservation, we subsampled enhancers with similar sequence constraints between the two classes and compared their degree of function conservation. Stable enhancers are significantly more conserved at the functional level compared to fragile enhancers, independent of the degree of sequence conservation (Figure S5).*”

**Figure S5 Comparisons of the two sets of enhancers with similar conservation levels of their fraction of functional conservation with mouse. The enhancers were partitioned based on their sequence conservation—the fraction of non-repetitive regions located within 46-way Plancental phastCon conserved elements. Fifty enhancers were sampled from each conservation bin for both classes and aligned to mouse; the process was repeated for 10 times. P-values were calculated based on Mann–Whitney U test. ** corresponds to p-value <= 0.005, * corresponds to pvalue <= 0.01.**

*3. P8 L28: "In addition, it has been observed that a significant number of genes involved in*

*cancer predate the origin of multicellularity, which is consistent with the strong conservation of stable enhancers". This is quite a severe overstatement: The fact that cancer genes are conserved across eukaryotes cannot be extrapolated to the conservation of their enhancers (see also Emera et al. PNAS 2016: genes with ancient functions are associated with mammal specific enhancers in the neocortex). Consistently, many of the cellular maintenance genes the authors associate with fragile enhancers are also very ancient, but this doesn't seem to be the case for their enhancers.*

We removed this statement from the manuscript, as the observed associations of stable

enhancers with cancer have not been explored in detail by our study and are not essential for the reported results.

*4. The authors interpret the weak effects of introducing deMs to their second construct*

*(human ortholog of mm69) as likely associated "with the enrichment of homotypic TFBS*

*clusters in this enhancer" (P12 L40). However, if I understand the CAPE method correctly,*

*adjacent TF binding and chromatin context is already taken into consideration when*

*determining deMs over non-deM mutations. Thus, this doesn't seem like a probable*

*explanation for the lack of striking differences between random and deMs in the same*

*enhancer. No classifier is expected to give perfect predictions and functional assays have a*

*considerable degree of biological variability, but the authors should be transparent about these limitations.*

We agree with the reviewer that the failure of the introduced deMs to completely abolish the activity of mm69 may due to the false positive predictions of CAPE, which to some extent might be caused by the slightly different regulatory codes between human and mouse, whereby the predicted deMs in human may not totally disrupt the binding of the same transcription factor in mouse. We have added this possible explanation to the manuscript (Page 13): “*We speculated that the failure of the introduced deMs to completely abolish the activity mm69 is possibly due to a false positive prediction of CAPE, which to some extent might be caused by the slightly different regulatory codes between human and mouse, whereby the predicted deMs in human may not totally disrupt the binding of the same transcription factor in mouse.*”

*Minor comments*

*1. P4 L7: "we observed that the deMP densities are correlated with the functional and*

*evolutionary stability of enhancers". I assume that the authors mean "negatively correlated"*

*instead.*

Yes, negatively correlated. Corrected.

*2. P13 L9: "Due to the high functional constraint on deMPs, fragile enhancers are prone to*

*one single nucleotide deactivation and, therefore, evolve faster". The authors should specify*

*that they refer to conservation of activity (rather than sequence).*

We have rephrased the sentence as “*Due to the high functional constraint on deMPs, fragile*

*enhancers are prone to single nucleotide deactivation and, therefore, are more likely to lose their regulatory function faster than stable enhancers during evolution.*”

*3. P14 L47: loose -> lose*

Corrected.

*4. Although the authors attempt to validate their deMs predictions in heart enhancers, all*

*other conclusions are exclusively supported by observations in HepG2 cells. However, this is a single cancer cell line, only remotely reflecting normal development. Thus, it is unclear whether these results can be generalized to enhancers active in primary cells or during organ development. This needs to be transparently acknowledged by the authors.*

We have added the following sentence to the end of Discussion to elucidate the limitation of our study: “*Nevertheless, the cancerous nature of the HepG2 cell line precludes us from fully generalizing our observations to enhancers active in primary cells.”*

**Reviewer #2:**

Overall, we found the manuscript reports an interesting analysis with potential broad

implications for enhancer biology. Nevertheless, we feel the manuscript needs to be

conceptually improved in several areas, specifically with additional analyses and discussion

points.

Major comments:

- It is unclear from the presented results whether stable and fragile enhancers may associate

with different levels of enhancer activity. Both ChIP-seq signal intensity and in vitro reporter

activity can be used as proxy measurements of enhancer activity, and there are several

published resources the authors could exploit to address this question (e.g. see (Kheradpour,

Ernst et al., 2013) and (Stefflova, Thybert et al., 2013)). Are stable enhancers also associated

with higher levels of enhancer activity compared to fragile enhancers?

Following the reviewer’s suggestion, we compared the regulatory activity of the three classes of

enhancers using several datasets available for the human genome: H3K27ac and H3K4me1

ChIP-seq signal intensity (Kundaje et al. 2015), liver-specific TF ChIP-seq signal intensity

(Bernstein et al. 2012; Gertz et al. 2013), and Sharpr-MPRA (Massively Parallel Report Assay)

enhancer activity score (Ernst et al. 2016).

We followed the procedure developed by the authors of Sharpr-MPRA to quantify the

regulatory activity of a region using MaxPos Sharpr-MPRA score (Ernst et al. 2016). For this

analysis, we used MaxPos Sharpr-MPRA region overlapping by at least 80% with the middle [-

200bp, 200bp] window of HepG2 enhancers, and used the MaxPos scores of these Sharpr-MPRA

sequences to quantify the regulatory activity of the three sets of HepG2 enhancers (Figure S12).

All the analyses of signal intensities of enhancer-specific histone marks (H3K27ac and

H3K4me1) and liver-specific transcription factors, and the Sharpr-MPRA scores indicate that

stable enhancers exhibit stronger enhancer activity compared to fragile enhancers, thus

validating the hypothesis suggested by the Reviewer (Figure S12).

These results have been added to the end of the first paragraph of Discussion (Page 14):

“*In addition, stable enhancers exhibit stronger enhancer activity compared to fragile enhancers, in*

*terms of H3K27ac ChIP-seq signal intensity, liver-specific transcription factor binding ChIP-seq signal*

*intensity, and in vitro regulatory activity measured by Sharpr-MPRA (Figure S12). Possibly, this is a*

*consequence of the larger number of functional and homotypic binding sites in stable enhancers, which*

*results in elevated stability of bound proteins and a diminished likelihood of transient binding.* ”

**Figure S12S. Stronger enhancer activity in stable enhancers. A) Stable enhancers show higher H3K27ac signal intensity. For enhancers with multiple H3K27ac peaks, only the peaks located closest to the enhancer center were included. B) Stable enhancers have greater Sharpr-MPRA regulatory scores. The number within parentheses below each boxplot gives the number of elements examined in Sharpr-MPRAs. C) Stable enhancers show stronger H3K4me1 signal intensity. D) Stable enhancers exhibit stronger TF binding ChIP-seq signal. P-values were calculated based on Mann–Whitney U test. * refers to P-value < 1e-3, ** refers to Pvalue < 1e-5.**

*The authors analyse sequence properties of stable and fragile enhancers by focusing on in silico prediction of transcription factor binding sites. While this approach is informative, it*

*obviates the known in vivo binding of transcription factors to a small sub-section of predicted*

*binding sites. Moreover, the authors' results suggest different modes of transcription factor*

*binding associate* with stable and fragile enhancers. These results would be strengthened by an

*analysis of transcription factor cooperativity in the two sets of enhancers, which could for*

*example leverage high-quality ChIP-seq data for liver-specific transcription factor binding in*

*mouse liver (Stefflova et al., 2013).*

This is a very important point. To address it, we leveraged all available 48 TF ChIP-seq datasets

available for the HepG2 cell line (unfortunately, we were not able to use the Stefflova et al.

mouse liver ChIP-seq dataset, as mapping of the mouse data onto the human HepG2 enhancers would likely have had a confounding effect of evolutionary differences between human and mouse TF binding). We analyzed TF-binding patterns in the three classes of HepG2 enhancers (stable, regular, and fragile enhancers; five-fold DHS regions were used as controls). The number of ChIP-seq peaks of a particular TF in a set of sequences was normalized by the total length of sequences in a dataset (Figure S6 below).

Consistent with the enrichment of *in silico* predicted TF motifs (Figure 4A), the stable enhancers are enriched for ChIP-seq peaks of FOXA1, FOXA2 and AP-1 (subunits: c-JUN, FOSL2), whereas the fragile enhancers are more enriched with binding of nuclear receptor factors HNF4A, HNF4G, and NR2F2 (Figure S6). This validates our reported differential enrichments of TFs in stable and fragile enhancers and confirms that the classes of TFs identified using an *in silico* approach are supported by the *in vivo* data analysis. In the revised version of the manuscript, the following statement has been added to reflect these new findings (Page 9): “*The enrichment of the in vivo transcription factor binding in the three classes of enhancers is consistent with the enrichment of in silico predicted TF motifs. Specifically, the stable enhancers are enriched for ChIP-seq peaks of FOXA1, FOXA2 and AP-1 (subunits: c-JUN and FOSL2), whereas the fragile enhancers show enrichment of nuclear receptor factors HNF4A, HNF4G, and NR2F2 (Figure S6).*”

**Figure S6: Different cohorts of TFBSs are enriched in enhancers with different densities of deMPs. The legend**

**shows the range of fold enrichment of TFBSs in a set of enhancers (random DHS regions used as background).**

**Only the TFs with the fold-enrichment >= 1.2 in at least one class of enhancers were included.**

Next, to compare the TF-TF interaction patterns in the two classes of enhancers using

ChIP-seq data, we first defined homotypic TF binding clusters [dubbed clusters of homotypic TF

binding regions (TFBRs)] as chromosomal segments that contain at least three TF ChIP-seq

peaks within a 1kb window. The same three TF ChIP-seq peaks per 1 kb definition was applied

to clusters of different TFs to define heterotypic clusters of TFs. Due to the lack of single

nucleotide resolution of TF ChIP-seq data (TF ChIP-seq peaks commonly span several hundred

bps and contain dozens of TFBS motifs), a single TF ChIP-seq peak could very well contain a

cluster (homotypic or heterotypic) of TFBS motifs and, thus, is not an ideal way for detecting

homotypic TF binding. As a consequence, only a tiny portion of enhancers harbor clusters of

homotypic TFBRs (Figure S18A). Therefore, the presented data analysis could be interpreted

only as a conceptual validation of the *in silico* clustering results, but not as a quantitative

comparison. Despite these limitations, the fold-enrichment analysis shows that the homotypic

ChIP-seq TFBRs are indeed enriched in stable enhancers, while heterotypic TFBRs are enriched

in fragile enhancers (Figure S18B; regular enhancers are used as a background for foldenrichment

computations).

**Figure S18. Cooperativity of TFs using ChIP-seq peaks. A) Number of clusters of homotypic/heterotypic TF binding regions (TFBRs) in the three classes of enhancers. B) Enrichment of TFBR clusters in stable (or fragile) enhancers relative to regular enhancers. The dashed line represents the background where fold enrichment = 1.**

**Figure 4D. Fragile and stable enhancers employ different regulatory codes. Clusters of homotypic TFBSs located within ChIP-seq peaks are more enriched in stable enhancers, whereas clusters of heterotypic TFBSs are more enriched in fragile enhancers. Only the TFBSs shown in Figure 4A were included in the analysis.**

Finally, to accurately quantify the TF-TF cooperativity pattern and capture the clusters of homotypic TFBRs supported by the ChIP-seq data, we identified *in silico* TFBS clusters (homotypic and heterotypic) using only *in silico* TFBS sites residing within their corresponding TF ChIP-seq peaks. For example, we would keep an HNF4a-predicted TFBS for this analysis only if it overlaps an HNF4a ChIP-seq peak. Our analysis shows that the stable enhancers are almost exclusively enriched for homotypic TFBS clusters, whereas heterotypic TFBS clusters dominate fragile enhancers (Figure 4D). This trend is consistent with the cooperativity patterns based on all potential TFBSs (Figure 4C) and further strengthens the conclusions of this study. Therefore we included this data into the main Figure 4 and added a following statement to the manuscript (Page 10): “*To further accurately quantify the TF-TF cooperativity pattern, we identified TFBS clusters (homotypic and heterotypic) using only the TFBS motifs residing within their corresponding TF ChIP-seq peaks. Our analysis shows that the stable enhancers are almost exclusively enriched for homotypic TFBS clusters, whereas heterotypic TFBS clusters dominate fragile enhancers (Figure 4D). This trend is consistent with the cooperativity patterns based on all potential TFBS motifs (Figure 4C).*”.

*Minor comments:*

*- It is not very clear from the results description in the main text whether the manuscript is solely focusing on distal enhancers, or whether the analysed regions also include proximal regulatory sequences. This is included in the supplemental methods but should be clarified in the main text, given their known differences in evolutionary conservation and stability. In this regard, the authors are focusing on HepG2 / liver enhancers and attributing an enrichment of liver-specific transcription factors to stable enhancers. This analysis is somewhat reductionist, and it may be useful to consider what is the observed tissue-specificity in the expression of the transcription factors the authors predict to be enriched in each set of enhancers. For instance, are stable enhancers also enriched for binding of ubiquitously expressed transcription factors, as could be expected from the evolutionary resilience of ubiquitously-expressed genes in the mammalian liver (Berthelot, Villar et al., 2018)?*

As the reviewer correctly highlights, our study is focused on distal enhancers and the proximal

promoter regions were removed from the analysis. Following the reviewer’s suggestion, we

added a clarifying statement to the Methods part of the manuscript (Page 18): “*Promoter regions*

*were defined as 1,500 base pairs (bps) upstream and 500 bps downstream from a TSS and were excluded from the set of enhancers.*”.

Next, we evaluated the tissue-specificity of the enriched TFs of the two sets of enhancers using the metric 𝜏 (Yanai et al. 2005), which ranges from 0 (consistent expression across all tissues) to 1 (expression limited to a single tissue/cell line). Gene expression profiles across 53 tissues were obtained from the GTEx portal (GTEx Analysis V7 release) on May 9, 2019. Consistent with the evolutionary resilience of ubiquitously-expressed genes in the mammalian liver (Berthelot, Villar et al., 2018), the TFs enriched in the stable enhancers exhibit a lower level of tissue-specificity compared to those enriched in fragile enhancers (Figure S13). The following statement has been added to the manuscript (Page 15): “*Interestingly, the TFs enriched in stable enhancers exhibit a lower level of tissue specificity compared to those enriched in fragile enhancers (Figure S13, Additional file 1), which is consistent with the evolutionary resilience of ubiquitously-expressed genes in the mammalian liver [32].*”

**Figure S13. Tissue-specificity (τ) of the TFs enriched in fragile enhancers (NR1I2, HNF4A, NR1H4, PPARG, NR4A1, PPARA, NR2F1, NR2C2, and NR2F6), and those enriched in stable enhancers (FOXA2, FOXA1, FOXD1, FOXO4, FOXO3, JUNB (AP-1), JUND (AP-1), FOSL2 (AP-1), and TCF7L2). The p-values were calculated based on Mann–Whitney U test.**

*- The concept of deMs, how they are predicted and what are the caveats in this computational approach should be clarified in the manuscript (i.e. to make it more accessible to nonspecialists).*

*This is a central concept in the study but is succinctly described in the main results section. It would also be useful to mention the background controls used in this method in the main results.*

As suggested by the reviewer, we have thoroughly introduced the basic idea and caveats of the

computational approach in the revised version of the manuscript (page 5): “*To identify the cisregulatory causal variants and examine their distribution in HepG2 cell line, we applied the SVM classifier CAPE [15], with the eQTL model (using random SNPs with matched minor allele frequency and similar distance to the nearest TSS as the negative control set) to predict mutations that are likely to affect gene expression and deactivate enhancers (Methods). The basic idea behind CAPE is to utilize a learned enhancer-associated sequence code to infer the deleterious effect of a potential regulatory genetic variant on enhancer activity. This approach integrates two characteristics associated with a genomic variation—the ability of a variant to disturb the cognate TF binding event and co-binding of other TFs in the neighborhood. These two sequence signatures were learned from a panel of enhancer-associated signals, including DNase-seq, H3K27ac, H3K4me1, H3K4me2, H3K4me3, H2A.Z, P300, and major TF binding data from the corresponding tissue [15] (Figure 1). In other words, the predicted deleteriousness of a genetic variation is composed of a weighted linear combination of the two signatures under a variety of enhancer-associated chromatin profiles. Therefore, a CAPE score can be further partitioned into two parts: a weighted linear combination of the binding affinity change across all signals [denoted as WS(*D*)] and a weighted linear combination of the neighborhood binding capabilities across all signals [denoted as WS(S)] (Methods, Figure 1). WS(*D*) and WS(S) represent the overall contribution of the binding affinity change and interactions of combinations of TFs within the surrounding sequence, respectively, to the impact on enhancer activity. The mutations identified by CAPE (FPR* <= *0.01) were dubbed deactivating mutations (deMs), as they are likely to disrupt the binding of an essential TF and lead to a deleterious impact on the enhancer activity and, in some cases, result in enhancer deactivation. The genomic positions holding at least one deM were dubbed deMPs. In general, this approach is limited to mutations that are observed frequently and that have matching expression observations, in the relevant tissue or cell type. For the tissues without eQTL information, this method is not directly applicable.*”

*- The authors should consider citing the primary publications from which they are leveraging published ChIP-seq data. The datasets and accession numbers are given in the Methods section,*

*but it would be useful to cite the relevant papers in the main manuscript for clarity.*

Thank you for noticing this oversight on our side. We are citing the corresponding original studies in the Data Access part of Methods in the revised version of the manuscript (Page 17): “*The H3K27ac ChIP-seq peaks of liver tissues in mouse, cow, dog and opossum originated from [36] were downloaded from EMBL-EBI ArrayExpress (accession number: E-MTAB-2633). The Hi-C contact map with 5-kb resolution in GM12878, HMEC, HUVEC, K562 and NHEK generated by [37] were downloaded from NCBI Gene Expression Omnibus (GEO) (accession number: GSE63525).*”

*- The authors may want to clarify / justify their focus on the two extreme sets of enhancers*

*(stable and fragile), as the "regular" category is largely omitted in the Figures. This is somewhat*

*covered in the supplemental info but would be useful to allude to in the main text.*

We have added the following statement to Results (page 6):“ *To test how deMPs in enhancers relate to the target gene function and the evolution of gene regulation in liver, we classified our set of HepG2 enhancers (n = 11,831) into three categories based on their deMP density: fragile enhancers (n = 2366, top 20%, 6.3% deMP nucleotides), regular enhancers (n = 2,366, middle 20%, 1.5% deMP*

*nucleotides), and stable enhancers (n = 2,366, bottom 20%, 0.25% deMP nucleotides) (Figure 2A, Figure S1). We speculated that the mutations occurring at deMPs might disrupt enhancer activity, while numerous deMPs would make an enhancer fragile, i.e., more prone to deactivating mutations.*

*Specifically, enhancers with more deMPs could lose their function during evolution faster compared to the ones with fewer deMPs. We primarily focused on two extreme sets of enhancers (stable and fragile) in this study. To investigate the relationship between the enhancer fragility and deMP density, we firstassessed the evolutionary constraints of deMPs by computing the percentage of the positions different from the chimp genome and the fraction of positions carrying single nucleotide polymorphisms (SNPs)…*”.

*- The format in some of the Figures needs to be revised:*

*- The scale in Figure 2D could be adjusted to the dispersion observed in the data. As*

*presented, it obscures the differences in medians across the three sets of enhancers.*

In addition to changing the scale of the Figure 2D, as suggested by the reviewer, we also

included the median values of the three classes in the figure.

*- Gene ontology terms in Figure 3A are unreadable. A vertical layout of this panel would aid readability*

Corrected.

*- The heatmap scale in Figure 4A could be annotated as fold change, again to helpreadability.*

Done.

*- The format and layout of Figure 5 should be improved (e.g. some of the axes labels are illegible).*

Corrected.

*- The discussion section could be improved in terms of balance and integration of the results with existing observations in the field:*

*- The discussion of tissue-specific transcription factor motif enrichment in stable enhancers (page 13, lines 47-54) somewhat obviates gene size as a potential confounder (i.e. transcription factor genes are larger than average, so more regulatory elements are associated to them and these may be larger on average). It is also unclear whether the authors analyses can correct for gene size.*

Contrary to the expectation, we observed that the gene locus length distribution is very similar for the two sets of enhancers, and the gene length doesn’t appear to be a contributing factor (Figure S11). In addition, stable enhancers are still longer than their fragile enhancer counterparts, even if we bin them according to the length of the associated gene locus (response figure). We added the following statement with the reference to this new figure to the revised manuscript (Page 14): “*Accordant with their observations, although the length of host gene loci have a similar distribution between the two sets of enhancers (Figure S11), the stable enhancers are longer (Figure S1) and harbor more functional elements (Figure 2D and Figure 4A) as compared to the fragile enhancers. This further suggests a stronger evolutionary constraint on the development-related enhancers (stable enhancers) compared to the immune system-related enhancers (fragile enhancers).*”

**Figure S11. Gene loci associated with fragile enhancers have length distributions similar to those associated with stable enhancers.**

**Response figure: The stable enhancers are longer than the fragile enhancers associated with similar length of gene locus. The genes were partitioned into five bins by their locus length.**

*- Many of the authors observations appear consistent with classic models of enhancer grammar (e.g. enhanceosome and billboard models). It would be helpful to include a discussion of these and related models (Spitz & Furlong, 2012) and how the observed properties of stable and fragile enhancers may relate to them.*

We have included a discussion of the linkage between the classical enhancer grammar models and the two classes of enhancers into Discussion (page 15): “*Therefore, the grammar of stable enhancers appears to be consistent with the billboard model, in which the positioning of TF binding sites is flexible and subject to loose distance, and only a subset of TFBSs are required to be active at a given*

*time [33, 34]. By contrast, the fragile enhancers are more likely to leverage heterotypic TFBS clusters and correspond to the enhanceosome model, in which the DNA motif composition and relative positioning act as a scaffold to cooperatively recruit all TFs, forming a higher-order protein interface to regulate transcription [34, 35]. In this scenario, all transcription factors that bind to an enhancer are essential for the cooperative occupancy and activation of an enhancer.*”

*- Minor revision of English language is needed throughout the manuscript.*

We carefully edited the revised version of the manuscript for grammar and spelling errors.

References:

Stefflova K, Thybert D, Wilson MD, Streeter I, Aleksic J, Karagianni P, Brazma A, Adams DJ,

Talianidis I, Marioni JC, Flicek P, Odom DT (2013) Cooperativity and rapid evolution of cobound

transcription factors in closely related mammals. Cell 154: 530-40

Berthelot C, Villar D, Horvath JE, Odom DT, Flicek P (2018) Complexity and conservation of

regulatory landscapes underlie evolutionary resilience of mammalian gene expression. Nature

ecology & evolution 2: 152-163

Kheradpour P, Ernst J, Melnikov A, Rogov P, Wang L, Zhang X, Alston J, Mikkelsen TS, Kellis M

(2013) Systematic dissection of regulatory motifs in 2000 predicted human enhancers using a

massively parallel reporter assay. Genome research 23: 800-11

Spitz F, Furlong EE (2012) Transcription factors: from enhancer binding to developmental

control. Nature reviews Genetics 13: 613-26

Stefflova K, Thybert D, Wilson MD, Streeter I, Aleksic J, Karagianni P, Brazma A, Adams DJ,

Talianidis I, Marioni JC, Flicek P, Odom DT (2013) Cooperativity and rapid evolution of cobound

transcription factors in closely related mammals. Cell 154: 530-40

Bernstein BE, Birney E, Dunham I, Green ED, Gunter C, Snyder M. 2012. An integrated

encyclopedia of DNA elements in the human genome. *Nature* **489**: 57-74.

Ernst J, Melnikov A, Zhang X, Wang L, Rogov P, Mikkelsen TS, Kellis M. 2016. Genome-scale

high-resolution mapping of activating and repressive nucleotides in regulatory regions.

*Nat Biotechnol* **34**: 1180-1190.

Gertz J, Savic D, Varley KE, Partridge EC, Safi A, Jain P, Cooper GM, Reddy TE, Crawford GE,

Myers RM. 2013. Distinct properties of cell-type-specific and shared transcription factor

binding sites. *Mol Cell* **52**: 25-36.

Kundaje A, Meuleman W, Ernst J, Bilenky M, Yen A, Heravi-Moussavi A, Kheradpour P, Zhang Z,

Wang J, Ziller MJ et al. 2015. Integrative analysis of 111 reference human epigenomes.

*Nature* **518**: 317-330.

Yanai I, Benjamin H, Shmoish M, Chalifa-Caspi V, Shklar M, Ophir R, Bar-Even A, Horn-Saban S,

Safran M, Domany E et al. 2005. Genome-wide midrange transcription profiles reveal

expression level relationships in human tissue specification. *Bioinformatics* **21**: 650-659.

**Second Round of Review**

**Reviewer 1**

Accept

**Reviewer 2**

The authors have done a careful job in addressing concerns raised by reviewers and the manuscript has been substantially improved. We detail below a few minor comments the authors may want to consider.

- Scientific English still needs substantial revision throughout the manuscript to improve clarity, precision and balance. Some specific examples:

- page 2, lines 11-12: “residing at the evolutionarily reliable foundation of development”. This sentence is somewhat impenetrable to the outside reader and rather subjective.

- page 4, line 24: “multiplicity of gene regulation mechanism”. Please reword to clarify meaning.

- often “genomic variation” is used where “genomic variants” would be the accurate wording (e.g. page 5, lines 7 and 11)

- page 7, line 12: “is the consequence of the multiplicity of deMPs”. The discussion of results in Figure S7 may need to be revised. If we understood correctly, these analyses aim to distinguish between sequence conservation and epigenomic conservation in fragile and stable enhancers, but cannot attribute causal effects.

- page 16, lines 6-8. Consider rewording to improve clarity

- page 16, lines 16-17: “as well as the motif variant locus”. Please reword to clarify meaning.

- As in some of the examples above, the interpretation of some of the study’s results should be revised and toned down. In particular, we are not convinced the authors can confidently exclude a role of TBFS density in influencing deMP density, given the caveats and limitations of the approach (Figure S7, page 10, line 8). These are correlative analyses and it would be helpful to present a more balanced interpretation. The same can be said of results in Figure 6CD (page 12, lines 15-16).

- The discussion of enhancer grammar models (page 15, lines 15-24) may need to be slightly revised. We agree with the authors that their results for stable enhancers are broadly consistent with the billboard model. However, the applicability of the enhanceosome model to fragile enhancers may need to be toned down: the authors do not analyse spacing constraints of TFBSs in these enhancers, and some of the classical enhancesome loci correspond to highly-constrained sequences. It may thus be more appropriate to reword some of their statements, for example along the lines of “fragile enhancers are more likely to harbour heterotypic TFBS clusters and may approximate the enhanceosome model, in which …”

**Authors’ responses to reviewers**

**Reviewer #1:**

**The authors have done a rigorous job at addressing my comments from the previous round of review, have further strengthened their manuscript with additional analyses and appropriately toned down their statements, where needed. In light of these changes, I would recommend the manuscript for publication.**

***Response: We would like to thank the reviewer for the positive comments about our work.***

**Reviewer #2:**

**The authors have done a careful job in addressing concerns raised by reviewers and the manuscript has been substantially improved. We detail below a few minor comments the authors may want to consider.**

**- page 2, lines 11-12: “residing at the evolutionarily reliable foundation of development”. This sentence is somewhat impenetrable to the outside reader and rather subjective.**

***Response: The sentence has been rephrased as “This dichotomy of enhancer activity is conserved across different tissues, has a specific footprint in epigenetic profiles, and argues for a bimodal evolution of gene regulatory programs in vertebrates. Specifically encoded stable enhancers are evolutionarily conserved and associated with development, while differently encoded fragile enhancers are associated with the adaptation of species.”***

**- page 4, line 24: “multiplicity of gene regulation mechanism”. Please reword to clarify meaning.**

***Response: We have reworded the sentence to “The goal of our study was to investigate the association between the density of causal regulatory variants in an enhancer, the DNA sequence composition and the evolutionary constraint of that enhancer.”.***

**- often “genomic variation” is used where “genomic variants” would be the accurate wording (e.g. page 5, lines 7 and 11)**

***Response: All the “genomic variations” in the manuscripts have been changed to “genomic variants” as suggested by the reviewer.***

**- page 7, line 12: “is the consequence of the multiplicity of deMPs”. The discussion of results in Figure S5 may need to be revised. If we understood correctly, these analyses aim to distinguish between sequence conservation and epigenomic conservation in fragile and stable enhancers, but cannot attribute causal effects.**

***Response: We agree with the reviewer and have rephrased the “the consequence of” as “largely associated with” on Page 7: “To investigate whether the lower functional conservation of fragile enhancers is largely associated with the multiplicity of deMPs apart from their lower degree of sequence conservation, we subsampled enhancers with similar sequence constraints between the two classes and compared their degree of function conservation.”***

**- page 16, lines 6-8. Consider rewording to improve clarity**

***Response: We have the modified the corresponding paragraph to “On the other hand, the usage of different TFBS collaboration modes in the two sets of enhancers may also corresponds to two alternative fashions to open the chromatin. Without the binding of pioneer factors to open the chromatin, the fragile enhancers have to recruit combinations of TFs to compete with nucleosomes to bind the DNA, sometimes in a manner of heterotypic TFBS clusters. In this regard, loss of binding of a TF due to one single nucleotide mutation might also lead to loss of binding of other synergistically bound TFs, inducing the deactivation of the enhancer. Conversely, the stable enhancers are enriched with the pioneer factor FOXA. Different from other enriched liver-specific TFs of stable enhancers, FOXA barely binds the enhancer in a mode of homotypic TFBS clusters (Figure 4E). Without multiple compensatory sites, the binding sites of FOXA that are prone to single nucleotide deactivation would have easily lost their function during evolution. Therefore, most FOXA binding sites are impervious to deMs in stable enhancers (Figure 4B), possibly by using a less constrained motif with shorter informative core motif compared to the FOXA binding sites that are more sensitive to deMs to some extent (Figure 5).”***

**- page 16, lines 16-17: “as well as the motif variant locus”. Please reword to clarify meaning.**

***Response: We have changed the statement to “As such, the majority of TFs can recognize regulatory regions based not only on pure sequence information of the motif, but also on the chromatin landscape of the DNA at neighboring sequences around the focal motif variant.” on page 16.***

**- As in some of the examples above, the interpretation of some of the study’s results should be revised and toned down. In particular, we are not convinced the authors can confidently exclude a role of TBFS density in influencing deMP density, given the caveats and limitations of the approach (Figure S7, page 10, line 8). These are correlative analyses and it would be helpful to present a more balanced interpretation. The same can be said of results in Figure 6CD (page 12, lines 15-16).**

***Response: We have rephrased the statements for more balanced interpretations as following:***

***On page 10, line 8: “We next examined the densities of these enriched TFBSs in the two categories of enhancers. Surprisingly, the two sets of enhancers have very similar densities of enriched TFBSs (Figure S7), so that TFBS densities might not be the cause of the different deMP densities. Therefore, it could be either the different TFBS composition or different inter-TF interactions that lead to the distinct deMP densities.”***

***On page 12, lines 15-16: “the interaction of TFs in the immediate vicinity or the larger chromatin context is likely to be a more important factor that governs the fragility of TF binding and enhancer activity (Figure 6CD).”***

**- The discussion of enhancer grammar models (page 15, lines 15-24) may need to be slightly revised. We agree with the authors that their results for stable enhancers are broadly consistent with the billboard model. However, the applicability of the enhanceosome model to fragile enhancers may need to be toned down: the authors do not analyse spacing constraints of TFBSs in these enhancers, and some of the classical enhancesome loci correspond to highly-constrained sequences. It may thus be more appropriate to reword some of their statements, for example along the lines of “fragile enhancers are more likely to harbour heterotypic TFBS clusters and may approximate the enhanceosome model, in which …”**

***Response: We have modified the statements as suggested by the reviewer on page 15: “By contrast, the fragile enhancers are more likely to harbor heterotypic TFBS clusters and may approximate the enhanceosome model, in which the DNA motif composition and relative positioning act as a scaffold to cooperatively recruit all TFs, forming a higher-order protein interface to regulate transcription [34, 35]. In this scenario, all transcription factors that bind to an enhancer might be essential for the cooperative occupancy and activation of an enhancer.”***
